# Protocol for an open-source system to integrate calcium imaging, pupillometry, and locomotion-estimated tracking in head-fixed mice

**DOI:** 10.1016/j.xpro.2024.103331

**Published:** 2024-09-30

**Authors:** Maria M. Ortiz-Juza, Jovan Tormes-Vaquerano, Sophia M. Hegel, Vincent R. Curtis, Rizk A. Alghorazi, Noah W. Miller, Ellora M. McTaggart, Nicolas C. Pégard, Jose Rodriguez-Romaguera

**Affiliations:** 1 Neuroscience Curriculum, University of North Carolina at Chapel Hill, Chapel Hill, NC 27514, USA; 2 Department of Psychiatry, University of North Carolina at Chapel Hill, Chapel Hill, NC 27514, USA; 3 Neuroscience Center, University of North Carolina at Chapel Hill, Chapel Hill, NC 27514, USA; 4 Department of Applied Physical Sciences, University of North Carolina at Chapel Hill, Chapel Hill, NC 27514, USA; 5 Department of Biomedical Engineering, University of North Carolina at Chapel Hill, Chapel Hill, NC 27514, USA; 6 Carolina Stress Initiative, University of North Carolina at Chapel Hill, Chapel Hill, NC 27514, USA; 7 Carolina Institute for Development Disorders, University of North Carolina at Chapel Hill, Chapel Hill, NC 27514, USA; 8 Department of Cell Biology and Physiology, University of North Carolina at Chapel Hill, Chapel Hill, NC 27514, USA

**Keywords:** Microscopy, Neuroscience, Behavior, Computer sciences

## Abstract

A wide selection of behavioral assays in systems neuroscience relies on head-fixation protocols to integrate *in vivo* multi-photon imaging approaches. For this, simultaneous pupillometry and locomotion tracking in head-fixed mice are used to measure behavioral responses and identify neural correlates. Here, we present an open-source protocol for assembling a complete head-fixation system that integrates pupillometry and locomotion-estimated tracking with multi-photon calcium imaging. We include detailed procedures for head-fixation and for data collection.

## Highlights

• Protocol for building a system for pupil and locomotion tracking in head-fixed mice
• Surgical procedure for implanting a head-ring with a GRIN lens for deep brain imaging
• Step-by-step instructions for habituating mice to a head-fixation system
• Turn-key GUI to integrate *in vivo* calcium imaging with pupil and locomotion tracking

## Abstract (editor highlights)

Publisher’s note: Undertaking any experimental protocol requires adherence to local institutional guidelines for laboratory safety and ethics.

## Graphical abstract

**Figure:**
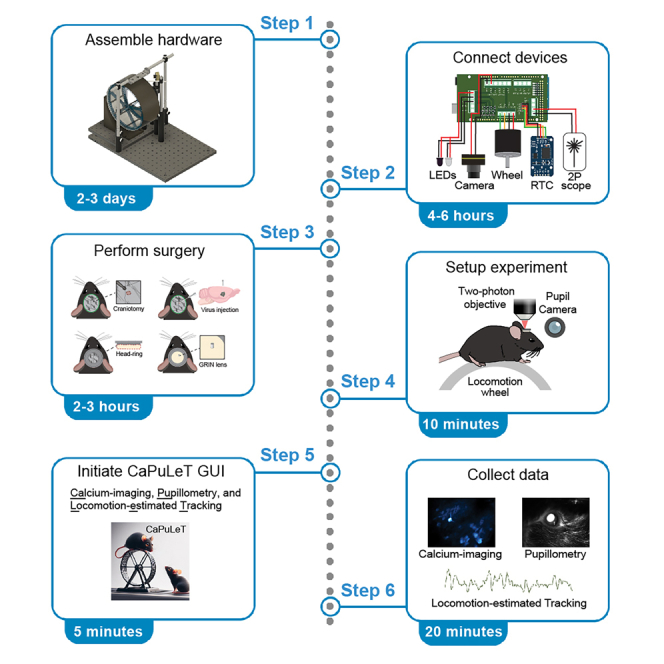

## Before you begin

**Figure 1:**
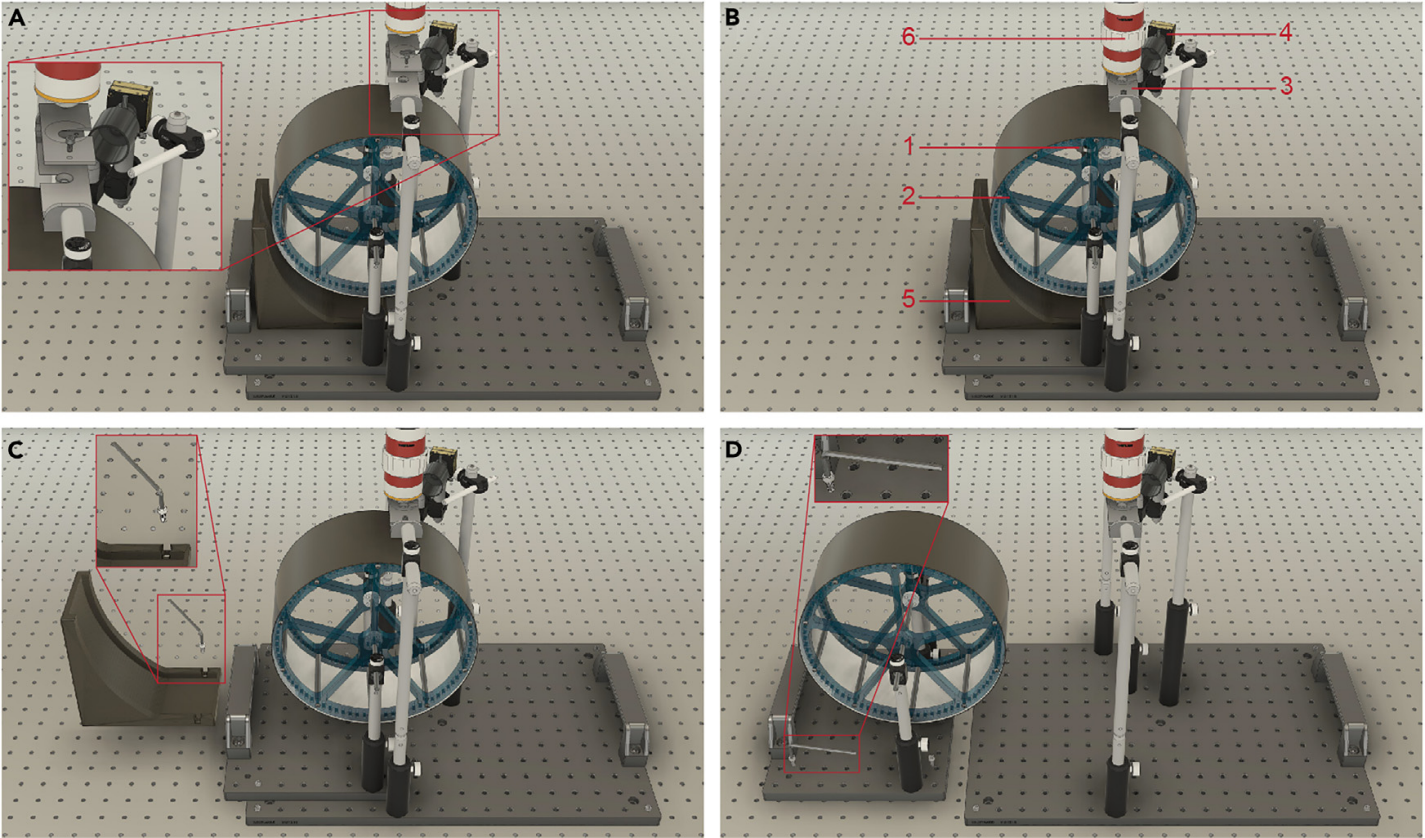
Head-fixation system with multi-photon imaging configuration (A) Prior to imaging, the objective of the multi-photon system can be raised (∼28 cm difference between air table and bottom of the objective) to provide more space to secure the head-ring between the headplates and ensure a comfortable animal posture. (B) During imaging, the distance between the objective and the head-ring can be adjusted to the imaging depths and the location of the focal plane beneath the implanted surface of the GRIN lens. The relative position of the (1) rotary encoder, (2) locomotion wheel, (3) headplate for head-fixation, (4) pupil camera, (5) waste management ramp, and (6) microscope objective is maintained for the duration of imaging. (C and D) After imaging, the waste management ramp and locomotion wheel can be detached from the main base to facilitate cleaning or system adjustments.

This protocol outlines the materials and methods needed to build a head-fixation locomotion wheel adapted for the integration of *in vivo* multi-photon imaging systems (Figures 1A–1D).^1^ Our technology is designed to simultaneously monitor pupil dynamics and body movements with an incremental (quadrature) rotary encoder and a pair of CMOS cameras. The frame of our system is built on an optical breadboard, allowing for adjustments of the wheel and headplates during head-fixation. An Arduino microcontroller, connected to a custom printed circuit board (Arduino shield), establishes a digital interface between all the devices in the system and the data acquisition computer. The Arduino shield includes a real-time clock module to provide precise time stamps for all the recorded data and to enable proper synchronization between recorded behavioral and neural data outputs. Additional external triggers on the Arduino shield are available to integrate other widely utilized behavioral or neural acquisition applications. The head-fixation system described in this protocol is a technological solution for parallel measurements of neural, behavior, and physiological data in awake behaving mice. In addition, we provide a turn-key MATLAB graphical user interface (GUI) along with editable scripts to enhance data collection and analysis efficiency, while offering the flexibility to adapt this interface to meet various research needs.

The step-by-step procedure for this protocol consists of the following: 1) assembly of the head-fixation system, 2) assembly of the Arduino printed circuit board, 3) preparation of aluminum head-rings for skull attachment, 4) surgical protocol for the implantation of gradient refractive index relay lens (GRIN) for calcium imaging in subcortical brain regions and for the attachment of the head-ring, 5) procedures to habituate mice to the head-fixation system, 6) extraction of pupil dynamics with DeepLabCut, and 7) synchronized data acquisition and collection during calcium imaging experiments.

### Institutional permissions

All procedures were conducted in accordance with the Guide for the Care and Use of Laboratory Animals, as adopted by the National Institute of Health, and with the approval of the Institutional Animal Care and Use Committee from the University of North Carolina at Chapel Hill.

### Fabrication of custom parts

**Timing: 2–3 weeks (dependent on lead times)**

**Figure 2:**
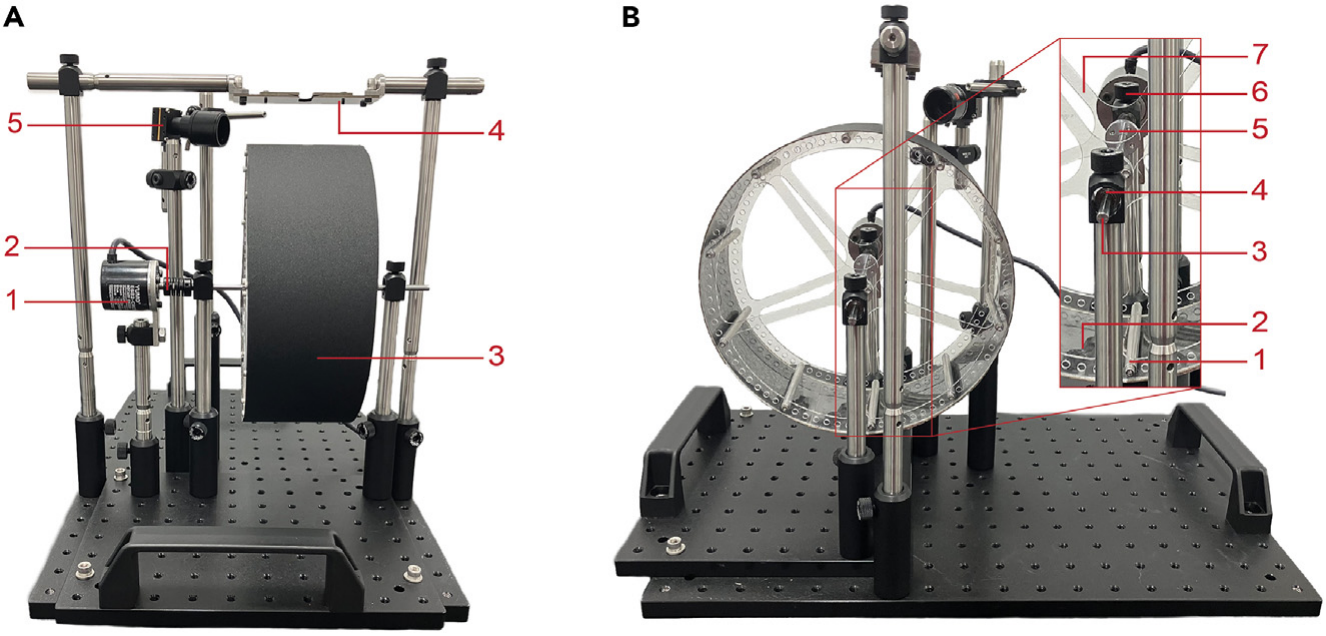
Custom parts of the head-fixation system (A) Back-to-front view of the assembled head-fixation locomotion wheel system, labeled to include the (1) rotary encoder, (2) rotary encoder adaptor, (3) locomotion wheel, (4) headplate for head-fixation, and (5) pupil camera. (B) Left-to-right view, labeled to include the (1) threaded round standoffs, (2) clear wheel surface, (3) linear motion shaft, (4) ball bearing, (5) mounting hub, (6) right-angle clamp, and (7) wheel spoke.

The head-fixation system is constructed from both commercial parts and custom-made parts (see the key resources table) (Figures 2A and 2B). The digital design files for 3-D printed parts, laser cut parts, printed circuit boards, and additional instructions for the preparation of these custom parts are available on GitHub: https://github.com/UNC-optics/CaPuLeT.

1. Clear cast acrylic cut to a diameter of 21.5 cm, with a 5 mm diameter center hole for the locomotion wheel.

***Note:*** The design of the locomotion wheel was inspired by previous work^2^ where stability of the laser cut wheel spokes are provided by aluminum standoffs and mounting hubs (Figure 2B). The combination of the laser cut wheel spoke design and lightweight materials used ensures that the mouse’s movements are accurately reflected by the wheel.

2. Clear polycarbonate film and black adhesive contact paper cut to a length of 67.56 cm and a width of 8.41 cm for the locomotion wheel.

***Note:*** The locomotion wheel surface consists of two layers (Figures 2A and 2B). The first, a flexible polycarbonate film which adheres to the wheel spokes with acrylic cement and provides a smooth surface for unimpeded locomotion. The second layer of contact paper provides additional traction for the animal, and can be easily cleaned, removed, and replaced after repeated use.

3. Stainless Steel 304 rotary encoder adaptor.

***Note:*** A 5 mm linear motion shaft is secured to the mounting hubs at the center of the wheel with a pair of set screws. Attached to the shaft, either side of the wheel, are 5 mm steel ball bearings that provide a smooth and stable rotation axis for the wheel and the encoder. The ball bearings are secured to a right-angle clamp mounted on a standard optical post. The rotary encoder adaptor aligns the encoder along the same plane of the linear motion shaft. These components are attached with a shaft coupler, allowing the wheel’s rotational movements to be registered by the rotary encoder (Figures 2A and 2B).

4. Aluminum 6061-T6 head-ring.

***Note:*** A 12 mm by 3.25 mm head-ring is surgically implanted on the skull of the mouse (See below: Figure 10). At the center of this cylindrical head-ring is a 5 mm center hole which serves as an imaging window (See below: Figure 9A). After implantation, the lightweight and compact design of the head-ring ensures that the mouse’s movement during non-experimental periods is unimpeded. The design of the head-ring was inspired by previous work.^3^^,^^4^

5. Stainless Steel 304 head-fixation plates and holders.

***Note:*** During habituation and experimental periods, two screws on the headplates can be tightened and used to secure the mouse at the top of the wheel (Figure 7B, step 5). The height of the headplates and wheel can be adjusted before use to increase head-ring stability and ensure a comfortable animal posture (Figure 1A).

6. 3D-printed resin waste management ramp.

***Note:*** A common concern during behavioral experiments is managing animal waste. Improper waste management can violate health and safety protocols related to biological waste and can induce stress for an animal which is prone to self-grooming. The 3-D printed waste ramp provided has two screw holes on the base, making it compatible with the optical breadboard that the head-fixation system is built on. Placed below the running wheel, the reservoir at the bottom of the ramp makes discarding and cleaning waste between experiments easier (Figure 1B).

7. Manufactured Arduino shield printed circuit board.

***Note:*** A custom Arduino shield attached to an Arduino microcontroller drives and powers all the electrical components of our head-fixation system (Figures 8B and 8C). After fabrication, the shield consists of resistors and LEDs. To complete the circuit, a series of N-channel MOSFETs, pin headers, and terminal screw blocks need to be soldered to the shield. The completed Arduino shield can receive commands from both Arduino IDE and MATLAB.

### Software setup

**Timing: ∼3 h**

**Figure 3:**
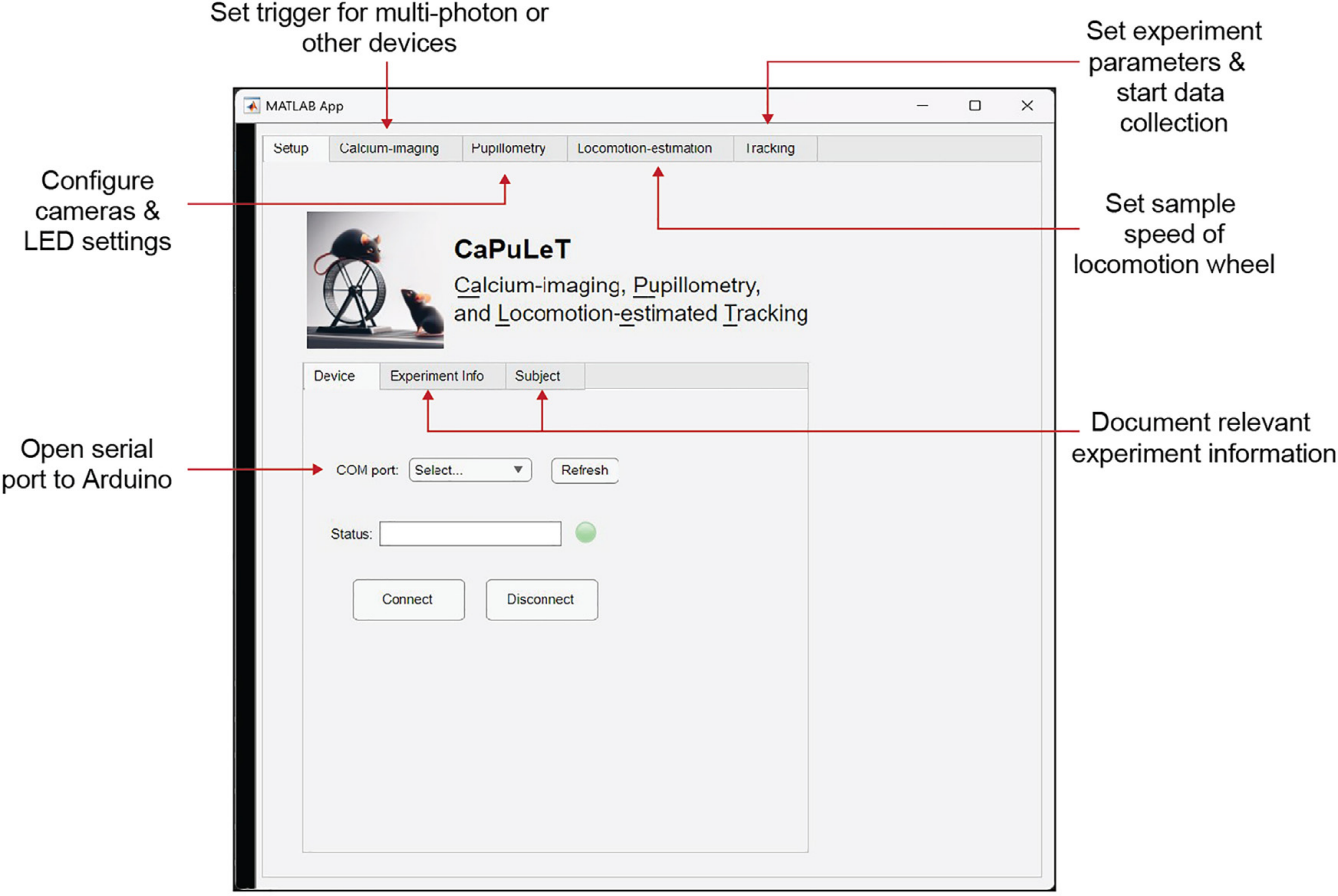
CaPuLeT (Calcium-imaging, Pupillometry, and Locomotion-estimated Tracking) Graphical User Interface CaPuLeT GUI layout when user launches program. Red labels indicate an overview of different capabilities found within each tab.

To ensure synchronized data acquisition in head-fixation experiments, our system relies on an Arduino microcontroller that receives serial instructions from a computer to control the devices connected to it. To configure and operate our system, we provide a user-friendly MATLAB GUI, named CaPuLeT (Calcium-imaging, Pupillometry, and Locomotion-estimated Tracking) (Figure 3). The GUI communicates with the Arduino and configures the camera for pupillometry measurements (see key resources table: Software and algorithms).

***Note:*** The data acquisition computers used in our experimental setup were equipped with a Nvidia 4070 Ti GPU, 64 GB of RAM, and an Intel i7-12700K. This configuration provided the necessary computational power to handle the real-time processing requirements of our calcium imaging and behavioral measurements. We recommend computers with at least 8 GB of RAM to run MATLAB and DeepLabCut.

8. Install the Spinnaker software development kit (SDK) for the pupil camera.
9. Install MATLAB version R2022b or newer.
  ***Note:*** Older versions of MATLAB may also be compatible with the CaPuLeT GUI, however, we have not tested this yet.
  a. Install the Image Acquisition Toolbox.
  b. Add hardware support packages for the pupil camera.
    i. Image Acquisition Toolbox Support Package for GigE Vision Hardware.
    ii. Image Acquisition Toolbox Support Package for GenICam Interface.
      ***Note:*** In our head-fixation system we use FLIR cameras to perform pupillometry; however, cameras from other manufacturers are equally capable of tracking pupil dynamics during head-fixation. MATLAB offers support packages that enable users to easily integrate other camera devices into our head-fixation system. It is important to note that some camera manufacturers (i.e. The Imaging Source) may require a different process to allow MATLAB to interface with that device (See troubleshooting: problem 1).
10. Connect pupil camera to a computer via USB 3.1 type-A to micro-B cable and test if camera is detected on MATLAB.

**CRITICAL:** If cameras are not listed under devices, but the support packages are installed, then the camera may not have the proper driver to be detected or other programs are accessing the camera (See troubleshooting: problem 2).

11. Install Arduino IDE.
  a. Install RTC library on Arduino IDE.
    i. Open Arduino IDE.
    ii. Navigate to Library Manager (Ctrl+Shift+I can be used to open manager).
    iii. Install NorthernWidget/DS3231 library.
12. Download and upload “CaPuLeT_Arduino_Script.ino” onto Arduino IDE.
13. Download and unzip *“CaPuLeT_GUI_Files.zip”.*

**CRITICAL:** For the GUI to operate properly, all files in the zip folder need to remain within the same folder and are not moved into different files or deleted.

### Notes on Arduino interface with wheel rotary encoder

**Figure 4:**
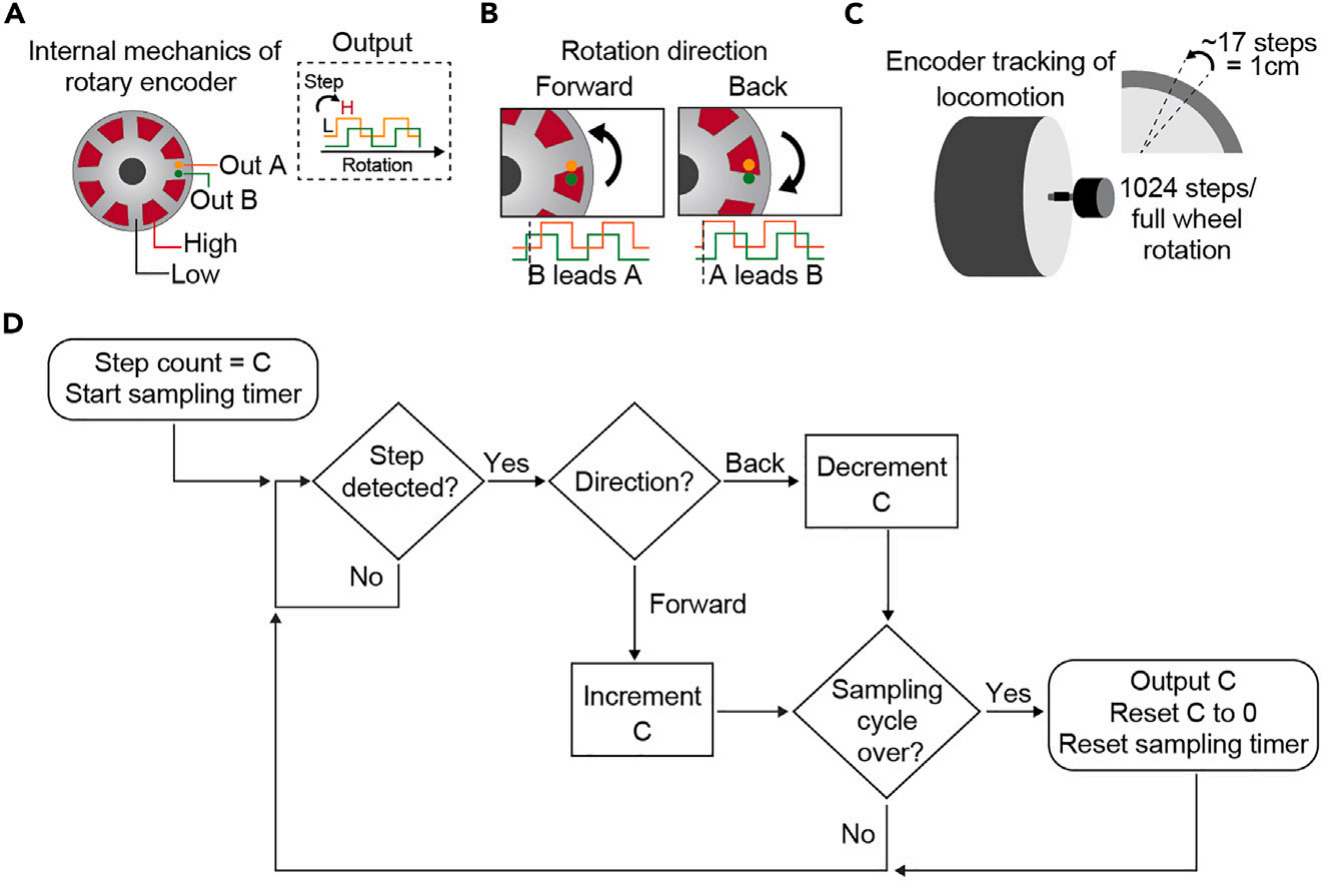
Incremental rotary encoder interface with Arduino (A) Schematic of the internal operation of an incremental rotary encoder which consists of two slightly shifted stationary pins (Out A and Out B) to detect voltage changes when the wheel is in motion. Right: Expected stepwise signal output from Out A and Out B pins. (B) Schematic of the two distinct signal outputs used to interpret rotational direction from the encoder. (C) Depiction of encoder bridged to wheel to track locomotion and the approximate metric to translate output from encoder to a unit of distance moved. (D) Block diagram explaining logic of interrupt-based interfacing code executed by the Arduino to count bidirectional rotations of the encoder.

Quadrature rotary encoders have two TTL channels (Out A, Out B) that output a binary signal when the wheel is displaced (XYZ positions per revolution) (Figures 4A and 4B). The rotary encoder also has an Out Z channel that sends a pulse when a full revolution on the wheel is reached. In our configuration, we do not use the Out Z channel. The resolution of the encoder indicates the number of displacements that the encoder can detect per full revolution. This value and the circumference of the wheel can be used to estimate velocity and distance traveled (Figure 4C). To ensure precise tracking of wheel movement, the Out A and Out B channel pins are attached to digital pins on the Arduino that will execute an interrupt service routine to count the number of positions displaced within a given sampling interval (Figure 4D). The Arduino then outputs this value to MATLAB.

### Notes on CaPuLeT GUI interface with Arduino

**Figure 5:**
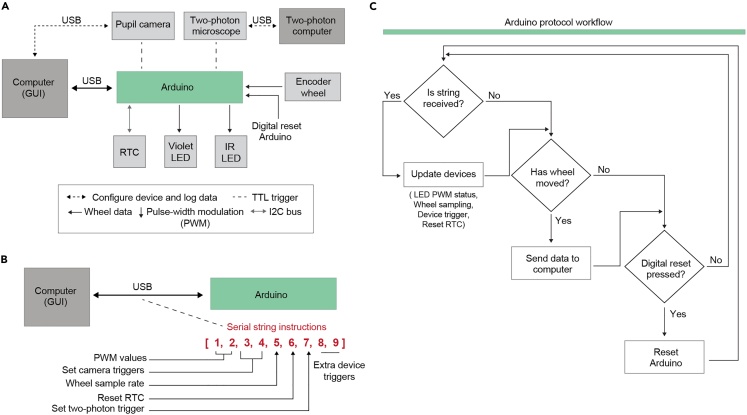
Arduino serial communication protocol to control device and data acquisition (A) Block diagram demonstrating the various connections each device in the head-fixation system has to the Arduino microcontroller and computers. The line type, width, or color within each connection indicates the operation that either device performs to configure components and collect data during experiments. (B) Schematic of string that contains instructions for devices that the Arduino receives from USB. (C) Block diagram of the order of operations the Arduino executes.

CaPuLet operates as an interface with Arduino to enable users to configure up to three device triggers to perform calcium imaging, optogenetics, etc., adjust camera settings for pupillometry, and set sampling speed for the locomotion wheel (Figure 3). To control devices and collect data during experiments, CaPuLeT transforms user input into a string of 9 numbers and sends that data to the Arduino via USB (Figures 5A and 5B). Once the Arduino receives the string, it executes a series of protocols to configure devices and to collect wheel data from the rotary encoder (Figure 5C). The values for numbers 1–2 in the string can range from 0 to 255 and set the pulse width modulation (PWM) for LED brightness. Numbers 3–4 and 7–9 range from 0 to 1 and encode whether camera, and calcium imaging or neural modulation devices will receive a TTL trigger to initiate data acquisition. Number 5 ranges from 0 to 1000 to set the sampling speed of the rotary encoder. To ensure device control and data acquisition is synchronized, we utilize a real-time clock (RTC) module. Therefore, number 6 ranges from 0 to 1 to send a TTL trigger to the RTC to reset time. This provides users with a timestamp to account for any drift that may occur during experiments.

## Key resources table

**Table:** 

REAGENT or RESOURCE	SOURCE	IDENTIFIER
**Chemicals, peptides, and recombinant proteins**		

Isoflurane	Covetrus	11695067772
Ophthalmic ointment	Dechra Veterinary Products	17033-047-05
Lidocaine topical ointment (5%)	Mohnark Pharmaceuticals, Inc.	73715-002-01
Triple antibiotic ointment	Trifecta Pharmaceuticals	69396-002-20
Nair hair removal cream	Local pharmacy	N/A
Saline (0.9% NaCl)	Hospira	0409-4888-02
Iodine solution (10%)	VetOne	13985-571-50
Ethanol solution (100%)	Decon Labs, Inc.	3916EA
Super Bond C&B Kit	Sun Medical	DCR176
Jet denture repair powder	Lang Dental	1230-FP
Jet liquid	Lang Dental	1404
Alloxate meloxicam (5 mg/mL)	Aspen Veterinary Resources	46066-937-13
Bupivacaine hydrochloride (0.5%)	Hospira	0409-1163-18
pAAV-EF1α-DIO-GCaMP6s (3.1 × 10^12^ infectious units/mL)	Addgene	105714

**Deposited data**		

Example wheel data	Pégard & Rodriguez-Romaguera Lab	https://github.com/UNC-optics/CaPuLeT
Example pupil data	Pégard & Rodriguez-Romaguera Lab	See GitHub

**Experimental models: Organisms/strains**		

Mouse: *Prepronociceptin*-IRES-Cre (male: 8 weeks)	Jackson Laboratory	034278

**Software and algorithms**		

MATLAB (version: R2023a)	MathWorks	https://se.mathworks.com/products/matlab.html
Arduino IDE (version: 2.3.2)	Arduino	https://www.arduino.cc/en/software
Spinnaker Software Development Kit (version 4.0.0.116)	Teledyne FLIR	https://www.flir.com/products/spinnaker-sdk/
DeepLabCut	Mathis et al.^5^	https://github.com/DeepLabCut
Custom R script for data analysis	GitHub	See GitHub
Custom graphical user interface	GitHub	See GitHub
Image Acquisition Toolbox (IAT)	MathWorks	https://www.mathworks.com/products.html
Data Acquisition Toolbox	MathWorks	https://www.mathworks.com/products.html
Image Processing Toolbox	MathWorks	https://www.mathworks.com/products.html
Statistics and Machine Learning Toolbox	MathWorks	https://www.mathworks.com/products.html
IAT Support Package for GenICam Interface	MathWorks	https://www.mathworks.com/products.html
IAT Support Package OS Generic Video Interface	MathWorks	https://www.mathworks.com/products.html
IAT Support Package for Point Grey Hardware	MathWorks	https://www.mathworks.com/products.html
IAT Support Package for GigE Vision Hardware	MathWorks	https://www.mathworks.com/products.html
MATLAB Support Package for Arduino Hardware	MathWorks	https://www.mathworks.com/products.html
Spinnaker SDK USB3 Vision Cameras Driver	Teledyne FLIR	https://www.flir.com/products/spinnaker-sdk/
IC Matlab Plugin for Matlab (version 3.4.0.58)	The Imaging Source	https://www.theimagingsource.com/en-us/support/download/icmatlabr2013b-3.4.0.58/
NorthernWidget/DS3231 RTC library	Wickert et al.^6^	https://github.com/NorthernWidget/DS3231

**Other**		

5 mm linear motion shaft	McMaster-Carr	6112K37
Clear impact-resistant polycarbonate film	McMaster-Carr	85585K15
Female Threaded Round Standoff	McMaster-Carr	93330A541
Socket head screw M3 × 0.5 mm thread, 6 mm long	McMaster-Carr	91290A111
Aluminum mounting hub for 5 mm shaft	Pololu	1203
Machine screw: #4–40, ½″ long (1.27 cm), Phillips	Pololu	1962
0.75 Inch (1.91 cm) post	Thorlabs	TR075
2 Inch (5.08 cm) post	Thorlabs	TR2
3 Inch (7.62 cm) post	Thorlabs	TR3
4 Inch (10.16 cm) post	Thorlabs	TR4
6 Inch (15.24 cm) post	Thorlabs	TR6
8 Inch (20.32 cm) post	Thorlabs	TR8
10 Inch (25.4 cm) post	Thorlabs	TR10
2 Inch (5.08 cm) post holder	Thorlabs	PH2
3 Inch (7.62 cm) post holder	Thorlabs	PH3
6 Inch (15.24 cm) post holder	Thorlabs	PH6
Right angle end clamp	Thorlabs	RA180
Rotating clamp	Thorlabs	SWC
Right angle post clamp	Thorlabs	RA90TR
Mini optical post	Thorlabs	MS3R
Aluminum optical breadboard 8″ × 10″ × ½″	Thorlabs	MB810
Aluminum optical breadboard 12″ × 18″ × ½″	Thorlabs	MB1218
Breadboard lifting handles, reinforced polymer	Thorlabs	BBH1
4–40 Stainless steel cap screw, 3/8″ long (0.95 cm)	Thorlabs	SH4S038
Spring-loaded 3/16″ hex-locking thumbscrew	Thorlabs	TS25H
¼″-20 Stainless steel setscrew, 5/8″ long (1.59 cm)	Thorlabs	SS25S0625
¼″ Washer, M6 compatible, stainless steel	Thorlabs	W25S050
¼″-20 Stainless steel nut	Thorlabs	N25S0440
Rotary encoder	Omron	E6B2-CWZ3E
Speaker 8 ohm 5 W top port 104 db	DB Unlimited	SP500208-3
2.54 mm Pitch single row pin header strip	SullinsCorp	PREC040SAAN-RC
MOSFET, SOT-23, 60 V, 0.28A, 150C, N-Channel	Diotec Semiconductor	2N7002
Standard LEDs, WL-SMCW SMT Mono-color Chip LED Waterclear 0805 Red	Wurth Elektronik	150080RS75000
Resistor, SMD 10K Ohms 0805 0.125W 1%	SEI Stackpole	RMCF0805FT10K0
Resistor, SMD 0805 anti-sulfur 0.5 W, 0.5%, 300 Ohm	Panasonic	ERJ-UP6D3000V
5 mm steel ball bearing	Boca Bearings	SMR115-ZZC #5 LD
9–22 mm 1/3″ F1.4 M12-mount lens with IR filter	Xenocam	XC0922LENS
USB 2.0 Type-A male to mini-B 5-pin male cable	Monoprice	844660054498
0.33 Inch (0.84 cm) varifocal lens 2.8–12 mm F1.3 Iris	Computar	TG4Z2813FCS-IR
3 mm UV light, 395–400 nm	Chanzon	100F3T-YT-WH-PU
5 mm super-bright IR LED, 940 nm	Adafruit	ADA388
24 AWG 50 feet electrical wire	Fermerry	Fermerry-39
Arduino Mega 2560 Rev3 microcontroller board	Arduino	A000067
USB 2.0 cable type A/B	Arduino	M000006
PCB Mount screw terminal block connectors	Tnisesm	CATN-T03B
Real-time clock module DS3231 AT24C32 IIC	Adeep	2pcsDS3231
3 V lithium battery	LiCB	CR2032
Heat shrink tubing	ELECFUN	RSG-KIT350
Black adhesive contact paper	Yancorp	707516138165
IPS Weld-On 3 acrylic plastic cement	Weld-On	TLLPN-21847771
12 V 5A DC power cable male female connector	CENTROPOWER	DCCORD-MF
12 V DC power connector barrel adaptor	CENTROPOWER	DCPLUG-MF
12 V Red LED flexible strip ribbon light	ALITOVE	AL5RWPBK12V
Power strip surge protector 10 ft.	Mifasopower	MFS311T
Anti-vibration pads	Sumato Stuff	SSHI00003
1″ × 12″ × 12″ Sound absorption wall panels	Focusound	FS0302A
4 inch (10.16 cm) Zip Ties with 18 lb tensile strength	TANTTI	604477847283
3M Scotch Super 33 + electrical tape – ¾ in × 52 ft	3M	MMM06133
Hirose 4 pin female HR10A-7P-4S to flying leads power cable	Alvin’s Cables	HR10A-7P-4S(73)
Dremel 3000-1/24 Variable Speed Rotary Tool Kit	Dremel	080596032555 043948095516
Breadboard jumper wires (male to female)	Antrader	QXAD0034
Firefly S camera	FLIR	FFY-U3-16S2M-S
Tripod adapter for Firefly S camera	FLIR	ACC-01-0017
USB 3.1 Locking cable	FLIR	ACC-01-2300
GPIO cable w/ 6 pins	FLIR	ACC-01-3015
USB 2.0 CMOS Monochrome camera	The Imaging Source	DMK-22BUC03
15U 35″ depth under desk soundproof server	Sysracks	SP15.900
Surgical tools: forceps, scalpel	RWD	SP0009-M
Isoflurane anesthesia system	RWD	R5001E
Feedback-controlled heating pad	FHC	40-90-8D
Stereotaxic apparatus	Kopf	Model 942
Cotton swabs	Puritan Medical Products	25-806 10WC
Insulin syringes	BD	305770
Standard surgical microscope	Leica	MZ 6
Gradient refractive relay lens (GRIN)	Inscopix	1050-004597
Microdrill	RWD	78001
Drill bits	RWD	78041
Wheel spokes	Custom made	N/A
Clear polycarbonate film wheel surface	Custom made	N/A
Adhesive contact paper wheel surface	Custom made	N/A
Rotary encoder adaptor	Custom made	N/A
Head-fixation top headplate	Custom made	N/A
Head-fixation bottom headplate	Custom made	N/A
Head-fixation headplate holders	Custom made	N/A
Head-ring	Custom made	N/A
Waste management wheel	Custom made	N/A
5 mm infrared LED	Custom made	N/A
Low-power 3 mm violet LED	Custom made	N/A
5050 SMD red LED strip	Custom made	N/A
Arduino shield printed circuit board	Custom made	N/A
Two-photon microscope	Olympus	FVMPE-RS
LCPLAN N 20× IR Objective (LCPLN20XIR)	Olympus	1-U2M445

## Step-by-step method details

### Assemble the head-fixation wheel system

**Timing: 2–3 days**

The mechanical components of the system are built in two sections: 1) locomotion wheel and 2) head-fixation headplates. Blueprints and labeled photos for each step of the construction process of the locomotion wheel, including how to build, mount, and secure it to the rotary encoder, can be found on our GitHub: https://github.com/UNC-optics/CaPuLeT.

1. Assemble the locomotion wheel.
  a. Build the base of the wheel (Figure 6A).
    i. Orient yourself to the two optical breadboards and consider the bottom-left most screw hole as coordinate (1,1).
    ii. Secure the small breadboard (20.32 cm × 25.4 cm × 1.27 cm) onto the large breadboard (30.48 cm × 45.72 cm × 1.27 cm) with four cap screws at positions (1,2), (1,11), (7,2), and (7,11).
    iii. On the small breadboard, insert set screws at positions (7,1), (7,7), and (7,9). Secure the handles with two cap screws at positions (1,3) and (1,9).
    iv. On the large breadboard, insert set screws at positions (7,1), (7,12), (8,9), (10,8) and (7,9). Secure the handles with two cap screws at positions (18,4) and (18,10).

**Figure 6:**
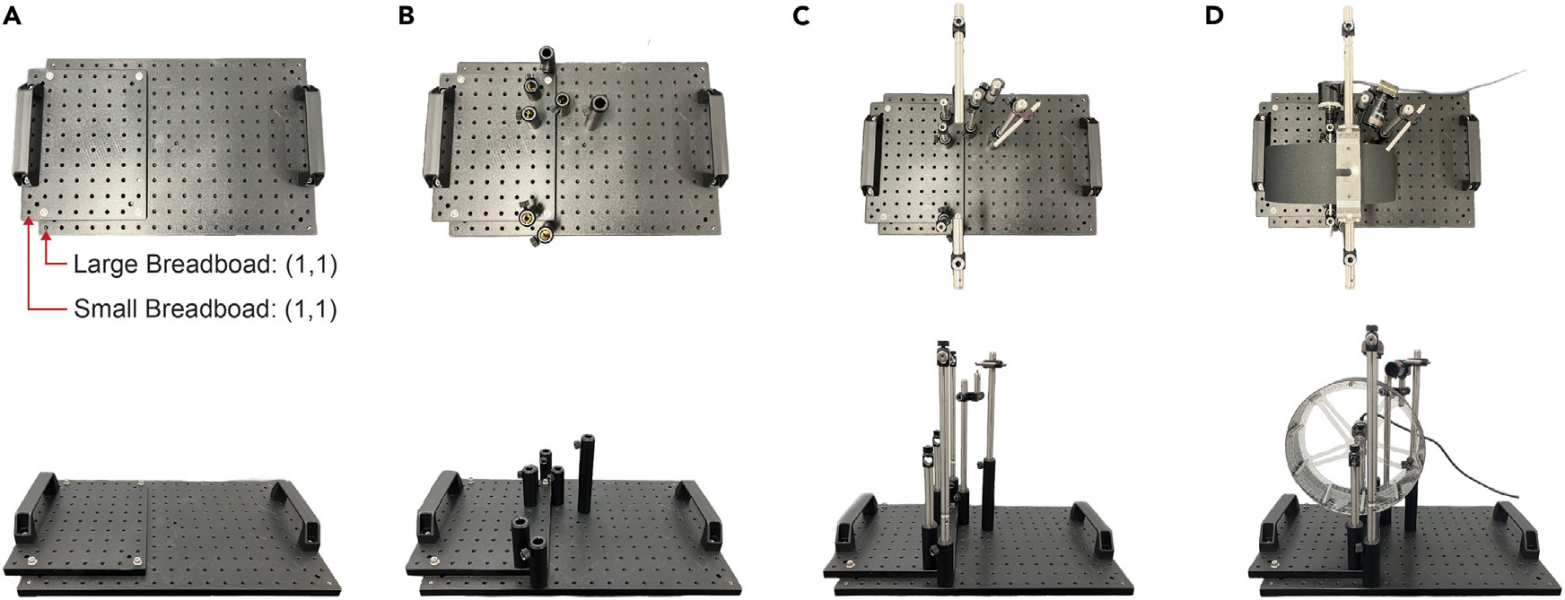
Assembly of head-fixation system (A) Top: Aerial view of the base of the head-fixation system. Red arrows specify the coordinate system for the Small and Large Breadboard. Bottom: Side profile of the base of the head-fixation system. (B–D) Aerial view and side profile of the head-fixation system after each successive level of construction.
  b. Connect the optical post holders (Figure 6B).
    i. On the small breadboard, screw optical post holders (Length = 7.62 cm) at positions (7,1) and (7,7) and optical post holder (Length = 5.08 cm) at position (7,9).
    ii. On the large breadboard, screw optical post holders (Length = 7.62 cm) at positions (7,1), (7,12), and (8,9), then optical post holder (Length = 15.24 cm) at position (10,8).
  c. Connect the optical posts (Figure 6C).
    i. On the small breadboard, insert optical posts (Length = 15.24 cm) at positions (7,1) and (7,7). Using a set screw, connect optical posts (Length = 5.08 cm and 7.62 cm) and insert at position (7,9).
    ii. On the large breadboard, insert optical posts (Length = 25.4 cm) at positions (8,9) and (10,8). Using a set screw, connect optical posts (Length = 10.16 cm and 20.32 cm) and insert at positions (7,1) and (7,12).
  d. Connect the right-angle end, right angle post, and rotating clamps (Figure 6C).
    i. On the small breadboard, screw right angle end clamps on the optical posts on positions (7,1) and (7,7), then secure a right-angle end clamp at position (7,9).
    ii. On the large breadboard, screw right angle end clamps on the optical posts on positions (7,1) and (7,12), then secure the rotating clamp at position (8,9) and the right-angle post clamp at position (10,8).
  e. Connect the remaining optical posts (Figure 6C).
    i. On the large breadboard, secure an optical post (Length = 7.62 cm) in the right angle end clamp at position (7,1). Using a set screw, connect optical posts (Length = 5.08 cm and 10.16 cm) and secure in the right-angle end clamp at position (7,12).
  f. Build the locomotion wheel.
    i. Laser cut two sheets of clear cast acrylic (Thickness = 0.312 cm) to produce two locomotion wheel spokes with a diameter of 21.49 cm.
    ii. Laser cut (or cut manually with a precision cutting tool) the clear polycarbonate film and black adhesive contact paper to a 67.56 × 8.41 cm rectangle.
    iii. Attach the mounting hubs to both wheel spokes using the 4–40 screws (Length = 1.27 cm) included.
    iv. Use 10 female threaded round standoffs to create a separation of 7.62 cm between the wheel spokes.
      ***Note:*** Align the standoffs to the spokes of the wheel and ensure that the mounting hubs are positioned on the inside wheel.
    v. Wrap the polycarbonate strip tightly around the edge of both wheel spokes and secure it with clamps and/or electrical tape.
      ***Note:*** Make sure that the polycarbonate strip is aligned without gaps or overlaps along both wheel spokes.
    vi. Apply acrylic plastic cement to both the outer and interior junction between the polycarbonate strip and wheel spokes.
    vii. Wait 12 h for the acrylic to dry, and carefully remove the clamps and/or electrical tape.
    viii. Apply the black adhesive contact paper to the outside of the polycarbonate strip.
      ***Note:*** A large section of contact paper can be applied to wrap the entire circumference of the polycarbonate strip on the outside of the wheel. A precision cutting tool can be used to trim along the edges of the wheel, ensuring smooth and complete coverage with the contact paper.
  g. Mount the locomotion wheel to the base (Figure 6D).
    i. On the small breadboard, replace the hex-locking thumbscrews (Length = 12.7 cm) of the right-angle end clamps at positions (7,1) and (7,7) with the longer spring-loaded hex-locking thumbscrews (Length = 17.1 cm).
    ii. Thread the 5 mm linear shaft through the wheel spokes and mounting hub and lock the shaft to the mounting hubs with the two set screws.
    iii. Slide the 5 mm steel ball bearings onto both ends of the shaft and insert the shaft through the right-angle end clamps.
    iv. Tighten the thumbscrews to secure the ball bearings in place, adjusting the position of the ball bearings accordingly to ensure alignment.
      ***Note:*** Longer spring-loaded hex-locking thumbscrews are required to securely fasten the 5 mm steel ball bearings. These ball bearings are secured to the linear shaft of the wheel and serve as the axis of rotation for the wheel, allowing the wheel to rotate smoothly.
  h. Connect the rotary encoder and locomotion wheel (Figure 6D).
    i. On the small breadboard, secure the rotary encoder adaptor to the right-angle end clamp at position (7,9) with a nut.
    ii. Secure the rotary encoder to the adaptor with three M3 screws (Length = 6 mm).
    iii. Insert the shaft of the rotary encoder to one end of the rotary encoder coupler, and the linear motion shaft attached to the locomotion wheel to the other end.
    iv. Tighten the set screws of the coupler to each shaft, creating a connection between the rotary encoder and the locomotion wheel.
      ***Note:*** After connecting the linear shaft and rotary encoder via the coupler, the locomotion wheel should rotate smoothly both clockwise and counterclockwise (See troubleshooting: problem 3). If there is resistance in spinning in either direction, the thumbscrews may be applying too much pressure to the ball bearings. After loosening, if any resistance remains, check to see if the optical post heights are even and whether the wheel itself is brushing against any component in the setup.
  i. Position the waste management ramp under the wheel.
    i. 3D-print the waste management ramp.
    ii. On the small breadboard, position the ramp directly underneath the locomotion wheel. Using 1 cap screw, secure the ramp to the breadboard. The position of this thumbscrew will depend on the position of the wheel (Figure 1B).
2. Assemble the head-fixation headplates (Figure 7A).
  a. Orientate the bottom headplate to the wheel.
    ***Note:*** Before constructing, ensure that the side of the bottom headplate with the semi-circular incision is facing the locomotion wheel. In this configuration, the bottom headplate’s flat side will align with the edge of the small breadboard.
  b. Connect the headplate holders (Figure 7B)
    i. With four M3 screws (Length = 6 mm), attach the headplate holders to the bottom headplate.
  c. Attach the headplate holder to the optical posts above the wheel.
    i. Align to the center of the wheel and secure the headplate holders to the two optical posts with two cap screws.
      ***Note:*** Aligning the head-fixation system to the wheel may require loosening the right-angle end clamps which hold the optical posts at positions (7,1) and (7,12) on the large breadboard. Once the center of the bottom headplate is positioned above the center of the wheel, in terms of width, you can tighten the headplate holders to the optical posts with the cap screws.
  d. Secure the head-ring between the headplates (Figure 7B)
    i. With two M3 screws (Length = 6 mm), attach the top headplate to the bottom headplate – securing the head-ring in place.
      ***Note:*** Prior to performing behavior experiments, test that each head-ring can be properly inserted in the bottom head plate (See troubleshooting: problem 4).
  e. Adjust the height of the head-fixation system and locomotion wheel.
    i. On the small breadboard, loosen the thumb screws of the optical post holders at positions (7,1), (7,7), and (7,9) to adjust the height of the locomotion wheel.
    ii. On the large breadboard, loosen the thumb screws of the optical post holders at positions (7,1) and (7,12) to adjust the height head-fixation system.
      ***Note:*** After completing prior steps, the head fixation system and locomotion wheel may need to be adjusted to accommodate a mouse.

**Figure 7:**
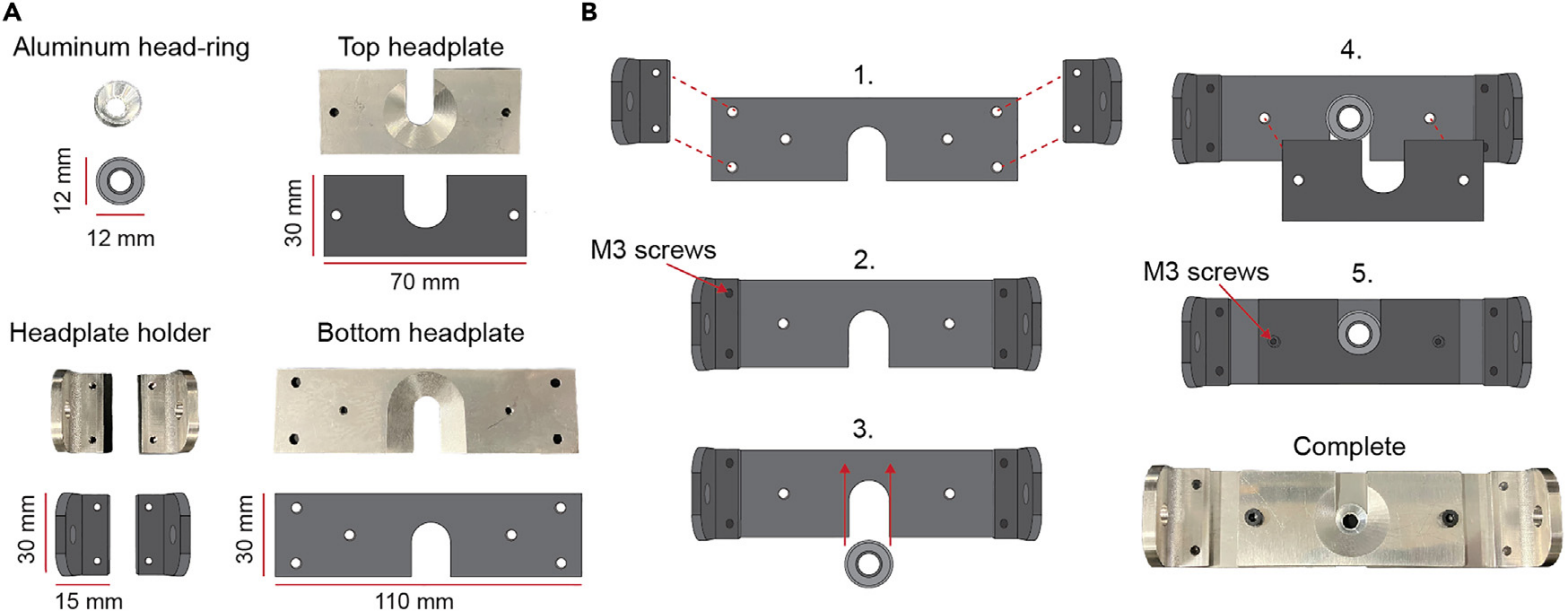
Assembly of head-fixation headplate (A) Diagrams outlining each of the four components of the head-fixation headplate, with accompanying dimensions. (B) Steps to secure the head-ring into the head-fixation headplate. Step 1: Align the headplate holders onto the bottom headplate. Step 2: Attach the holders to the bottom headplate with four M3 screws. Step 3: Insert the head-ring into the grooved section of the bottom headplate. Step 4: Align the top headplate onto the bottom headplate. Step 5: Secure the head-ring between the top and bottom headplates with two M3 screws.

### Assemble the Arduino shield printed circuit board

**Timing: 4–6 h**

**Figure 8:**
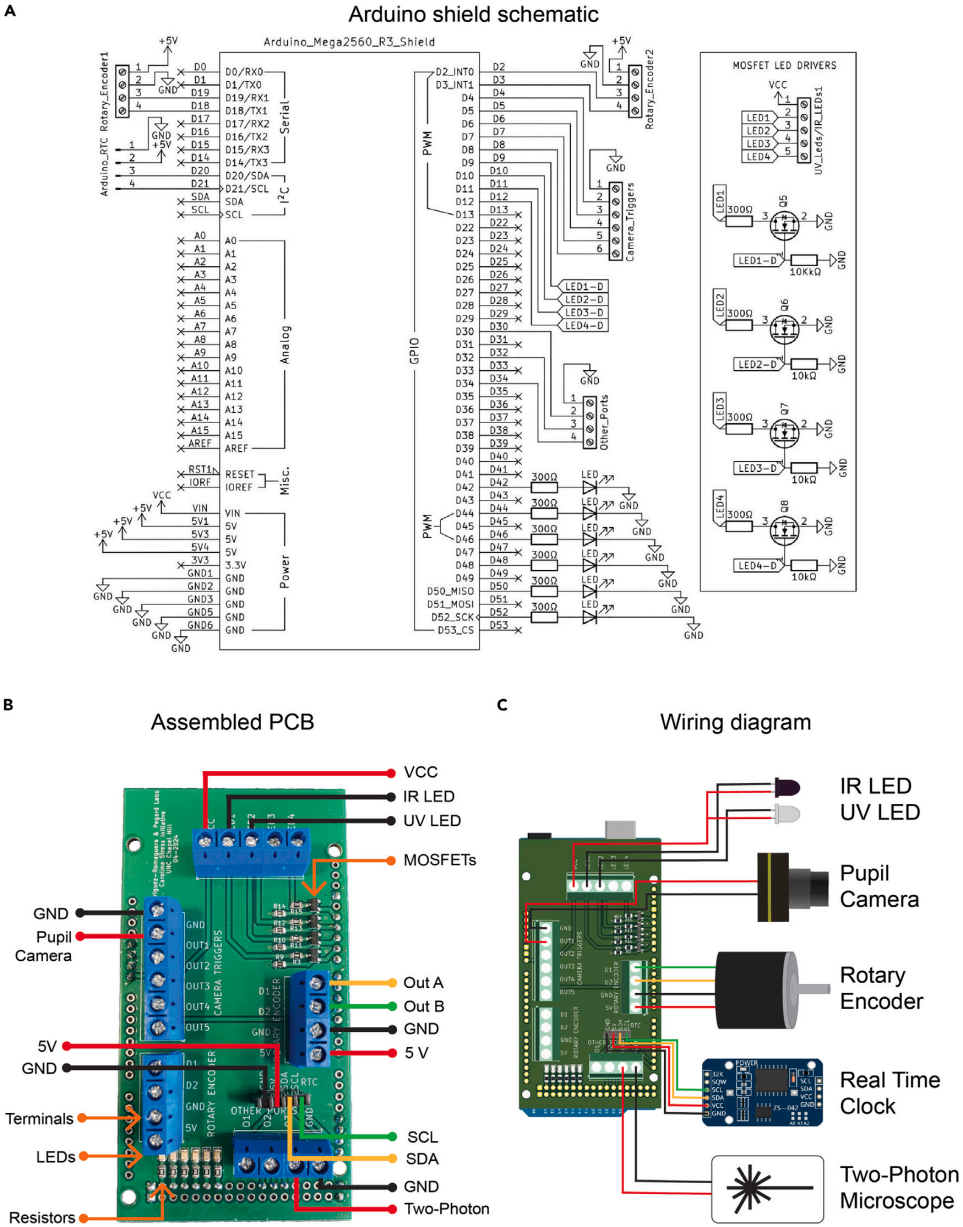
Arduino shield circuit and wiring diagrams (A) Schematic of printed circuit board diagram. (B) Image of the assembled printed circuit board with N-channel MOSFETs, resistors, LEDs, and terminal screw blocks soldered to the board. Each of the labeled wires are necessary to power and trigger a single head-fixation system. (C) Wiring diagram to connect rotary encoder, LEDs, cameras, and RTC module to the Arduino microcontroller.

Here we describe the steps to assemble the custom-made Arduino shield printed circuit board (Figure 8A). Within GitHub, we provide all the relevant fabrication, assembly, and Gerber files for this printed circuit board. Our design incorporates larger 0805 packaging for surface mount technology (SMT) and through-hole technology (THT) for electronic components. The surface mount device (SMD) MOSFETs, resistors, and LEDs can either be soldered manually using a soldering iron, soldered simultaneously using a reflow oven, or ordered assembled from a PCB manufacturer. In this section, we also describe how to solder each circuit element to the printed circuit board and how to connect the LEDs, rotary encoder, pupil camera, and two-photon microscope. Detailed schematics and further information on the assembly of the Arduino shield is available on GitHub: https://github.com/UNC-optics/CaPuLeT.

***Optional:*** If you choose to use a breadboard instead of the Arduino shield, you will need to provide resistance to avoid a short circuit. Instead of recreating the N-channel MOSFET configuration in (Figure 8A), you can solder resistors between the red wire and the anode of the LEDs. However, the MOSFETs in the current configuration allow you to actively control the current. Resistors are passive elements, and don’t provide a mechanism to amplify the current, or to switch it on and off.

3. Solder circuit elements onto the Arduino shield.
  a. Align and solder the N-channel MOSFETs.
    **CRITICAL:** Compared to the THT pin headers and terminal screw blocks, all SMD electrical components have flat, metallic pin pads which make electrical contact with the PCB. When soldering, the orientation of both the MOSFETs and LEDs are important for function. Identify the anode and cathode on the SMDs before soldering.
  b. Align and solder the terminal screw blocks (Figure 8B).
    i. Insert terminal screw blocks in each of the 23 locations on the Arduino shield, made from joining together 2-pin and 3-pin wide blocks.
    ii. Apply solder to the copper rings surrounding each pin and heat with the soldering iron until it melts, and the pins are secured onto the shield.
  c. Identify the male header pin locations and solder.
    i. Insert a male pin header in each of the 33 designated locations on the Arduino Microcontroller (Figure 8A).
    ii. Place the shield on top of the Arduino microcontroller, such that the pin headers are visible through the copper rings of the shield.
    iii. Repeat step 3b, section ii.
  d. Identify the female header pin locations and solder.
    i. Insert a female pin header in each of the 4 locations associated with the real- time clock (RTC) module (Figure 8B).
    ii. Repeat step 3b, section ii.
  e. Connect the RTC module to the Arduino shield (Figure 8B).
    i. Connect the right-angle header pins of the RTC module labeled power (VCC), ground (GND), Serial Clock (SCL), and Serial Data (SDA) to their respective female header pin (Figure 8C).
4. Construct and test the 5 mm infrared LED and low-power 3 mm violet LED.
  a. Prepare wires of opposing color and sufficient length.
    i. Prepare two wires of opposing colors. For our system, we used red and black wire for power and ground, respectively.
      ***Note:*** The length of the wires soldered to the infrared and violet LEDs, and all subsequent wired cables, will depend on the scale of the experimental setup. Specifically, the distances between the locomotion wheel, the Arduino microcontroller, and the data acquisition device. In our experimental setup, the wires soldered to the infrared and violet LEDs are 182 cm. This is identical to the length of the rotary encoder’s connector cable.
      ***Note:*** The microcontroller is positioned outside of the sound-proof box, with the wires for the infrared LED, violet LED, and rotary encoder routed into the box. When powered on or reset, the microcontroller will emit light from a pair of built-in red LEDs. To maintain a light-free environment within the sound-proof box during experiments, the microcontroller and additional electronic components are kept outside the box to minimize potential sources of noise.
    ii. Use wire strippers to strip both ends of the wires, exposing approximately 6 mm of wire.
    iii. Twist both ends of the wire into a single strand and apply a small amount of solder to avoid fraying and breakage.
      ***Note:*** This process is called tinning, and the resulting wires are referred to as tinned wires.
  b. Solder one wire to the anode and the other to the cathode.
    i. For both LEDs, wrap the tinned red wire around the anode of the LED, which is the lead that is slightly longer.
      ***Note:*** If you choose to use a breadboard instead of the Arduino shield, you will need to provide resistance to avoid a short circuit. If you don’t want to recreate the N-channel MOSFET configuration in (Figure 8A), you can solder resistors between the red wire and the anode of the LEDs. However, the MOSFETs allow you to actively control the current. Resistors are passive elements, and don’t provide a mechanism to amplify the current, or to switch it on and off.
    ii. For both LEDs, wrap the tinned black wire around the cathode of the LED.
    iii. Solder the wires to the LED leads, creating an electrical connection, and insulate the area of exposed metal with electrical tape or heat shrink tubing.
  c. Establish a connection between the LEDs and the Arduino shield.
    i. Connect the remaining tinned red wire (V_in_) of the LEDs into the same designated screw terminal of the Arduino shield (Figure 8B).
    ii. Connect the remaining tinned black wire (GND) of the LEDs into the different designated screw terminals of the Arduino shield (Figure 8B).
5. Construct and test the 5050 SMD red LED strip.
  a. Strip the protective rubber to expose the solder pads.
    i. The 5050 SMD red light strip has an LED approximately every 1.67 cm. Cut a long enough strip to illuminate your working area within the sound-proof box with red light.
    ii. Repeat step 4a to construct tinned wires capable of connecting the red LED strip to the female barrel adaptor.
    iii. Using a precision cutting tool, strip away the silicone coating on the LED strip to expose the anode and cathode solder pads.
  b. Solder the wires to the anode and cathode pads of the LED strip.
    i. Place the tinned red wire onto the anode solder pad, designated with a plus (+) sign.
    ii. Place the tinned black wire onto the cathode solder pad, designated with a negative (−) sign.
    iii. Solder the wires to solder pads of the LED strip, creating an electrical connection, and insulate the area of exposed metal with heat shrink tubing.
  c. Connect the female barrel adaptor to a 12V DC power connector.
    i. Connect the remaining tinned red wire (V_in_) of the LED strip into the anode port of the barrel adapter, designated with a plus (+) sign.
    ii. Connect the remaining tinned black wire (GND) of the LED strip into the cathode port of the barrel adapter, designated with a minus (−) sign.
    iii. Connect the barrel adaptor to a 12 V DC power connector.
    iv. Test the LED strip by connecting the power connector to a wall outlet.
6. Construct and test the rotary encoder
  a. Strip the insulating rubber from the unterminated end of the rotary encoder cable, providing access to the aluminum shielding and the five exposed wires.
    ***Note:*** While there are five exposed wires, only four are required for rotary encoder function in the system. The brown wire (+5 V DC), blue wire (0 V DC), black wire (phase A), and white wire (phase B) are necessary for this system. The orange wire (phase z) is an identical circuit to the black and white wire. The blue wire (0 V DC) should be connected to an external ground.
  b. Repeat step 4a, section ii and iii. Connect 4 tinned wires of the rotary encoder cable to the designated screw terminals of the Arduino shield (Figure 8B).
7. Connect and test the pupil and additional cameras.
  a. Establish a connection between the pupil camera and the computer.
    i. Connect the camera to the data acquisition computer via a USB 3.1 type-A to micro-B cable.
    ii. Open the SpinView application. The camera should be available on the device menu.
      **CRITICAL:** If the camera is not accessible on SpinView but listed on the computer’s Device Manager, then it is likely the camera was configured with the wrong driver (See troubleshooting: problem 1). It is important that the camera is available on SpinView for future steps (See troubleshooting: problem 2).
  b. Establish a connection between the pupil camera and the Arduino shield.
    i. Prior to connecting the GPIO cable to the pupil camera, repeat step 4a to extend its range if it cannot reach the Arduino shield.
    ii. Connect the tinned orange wire (+5 V DC) and tinned brown wire (GND) of the GPIO cable to the designated screw terminals of the Arduino shield (Figure 8B).
    iii. Insert the 6-pin male JST connector of the GPIO cable to the pupil camera.
  c. Establish a connection between the additional camera and the computer.
    i. Connect the camera to the data acquisition computer via a USB 2.0 type-A to mini-B cable.
    ii. Open MATLAB and select the correct camera from the Imaging Acquisition Explorer application menu.
  d. Establish a connection between the additional camera and the Arduino shield.
    i. Strip the insulating rubber from the unterminated end of the Hirose cable, providing access to the aluminum shielding and the four exposed wires. Repeat step 4a, section ii and iii.
    ii. Connect the tinned wires of the Hirose cable to the designated screw terminals of the Arduino shield (Figure 8B).
      ***Note:*** The input/output configuration for this 4-pin Hirose cable is as follows: red wire (pin 1, external ground), white wire (pin 2, open drain), green wire (pin 3, reference ground), and black wire (pin 4, trigger input). The red and green wires, associated with pins 1 and 3, are connected to ground pins of the Arduino microcontroller. The white and black wires, associated with pins 2 and 4, are connected to the digital output pins of the Arduino microcontroller.
    iii. Connect the 4-pin female connector of the Hirose cable to the additional camera.
8. Attach the camera lens to the pupil camera.
  a. An IR filter lens is required to record pupil dynamics. Screw the IR filter lens to the pupil camera (Figure 1A).
  b. Adjust the focal length by loosening either of the two thumb screws and twisting the lens. Twisting the two separate barrels of the lens adjusts the focal length between 9 mm and 22 mm.
9. Remove the shutter of the camera lens, then attach it to the additional camera.
  ***Note:*** The additional camera also requires an IR filter lens, however prior to attaching the filter we must remove the lens shutter. Given that we are recording continuous videos, the motorized lens shutter isn’t required.
  ***Note:*** The lens shutter is built into the body of the lens. To access you first need to unscrew the two thumb screws, then unscrew the three screws on the rear lens cap and the two screws on the body of the lens.
  a. Remove the rear lens cap, setting aside both the lens cap and the metal cs-mount underneath it.
  b. Holding the 4-pin male wire coming from the lens, remove the lens barrel. This will expose the GPIO cable connecting the lens shutter to the 4-pin male wire.
  c. The lens shutter is secured to the lens with two screws found near the base of the GPIO cable. After unscrewing, the lens shutter can be removed.
  d. Work backwards from steps 9c to 9a to rebuild the camera lens. Attach the rebuilt IR filter lens to the additional camera.
  e. Adjust the focal length by loosening either of the two thumb screws and twisting the lens. Twisting the two separate barrels of the lens adjusts the focal length between 2.8 mm and 12 mm.
10. Configure the Arduino microcontroller.
  a. Insert and tighten the wires of each component into the Arduino shield.

***Note:*** At this point, all principal components should be secured to the Arduino shield. If not, refer to steps 4c, 6b, 7b, and 7d.

### Prepare aluminum head-ring for skull attachment

**Timing: <30 min**

**Figure 9:**
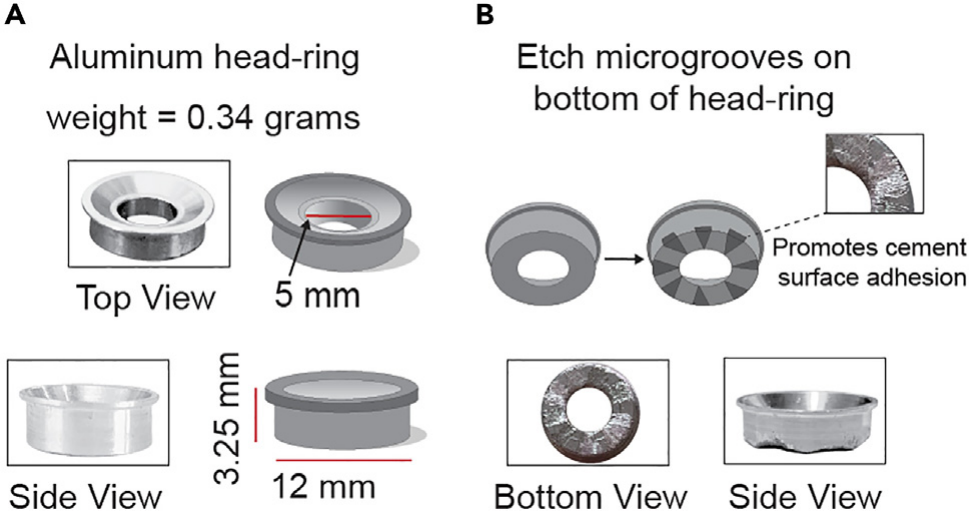
Preparation of aluminum head-ring (A) Weight and dimensions of aluminum head-ring attached to mouse skull for head-fixation. (B) Results of the microgrooves etched on bottom of head-ring to promote head-ring adhesion to mouse skull.

Reducing noise artifacts during mouse movement is important for obtaining optimal data in calcium imaging experiments. To achieve stable head fixation and restrict head movement during experiments, we utilize a lightweight aluminum head-ring (Figure 9A) designed for maximum mechanical stability without hindering mouse movement during non-experimental periods.^1^^,^^3^^,^^4^ Here, we outline the process of mechanically etching the underside of the head-ring to enhance stability and adherence to the mouse skull.

11. Clean bottom surface of the aluminum head-ring and outline ∼8 evenly spaced locations that will be etched (Figure 9B).
12. Secure the head-ring in a clamp or other tool to prevent it from moving during the etching process.
13. Select tools for the head-ring etching process.

***Note:*** Any high-speed metal cutter can be used; however, we use a Dremel 3000.

***Note:*** If using a Dremel, select a small Dremel bit that will allow etching of the 8 locations. In addition, the material for the Dremel bit should also be good for grinding and carving on harder materials, such as an abrasive stone.

**CRITICAL:** Prior to initiating the etching process, ensure proper safety precautions. Wear appropriate personal protective equipment, including safety glasses and a dust mask to protect yourself from metal shavings and dust generated during the etching process.

14. Carefully etch the head-ring until small microgrooves are made (Figure 9B).

***Note:*** The small microgrooves will allow more surface for the dental cement to adhere the head-ring onto the skull.

15. Thoroughly clean the head-ring to remove any remaining debris or dust.

### Surgically implant GRIN lens and head-ring

**Timing: ∼3 h/mouse**

Here, we describe the surgical procedure required to perform two-photon calcium imaging in head-fixed mice. The main steps we follow are based on standard surgical practices used for calcium imaging studies.^7^^,^^8^^,^^9^^,^^10^ These include viral injection of genetically encoded calcium indicator GCaMP6s, implantation of GRIN lens (0.6 mm in diameter, 7.3 mm in length), and surgical attachment of the head-ring. The time range from post-surgery to experiment ranges from 4–6 weeks to account for recovery of mouse and sufficient viral transduction. In addition, the procedure and drugs used in this protocol should be adapted according to approved IACUC protocols.

***Note:*** In this protocol, we implant and affix the head-ring for studies focused on subcortical brain structures located near bregma. Studies focused on brain regions outside of this range can update the head-ring shape and dimension file located on GitHub.

**Figure 10:**
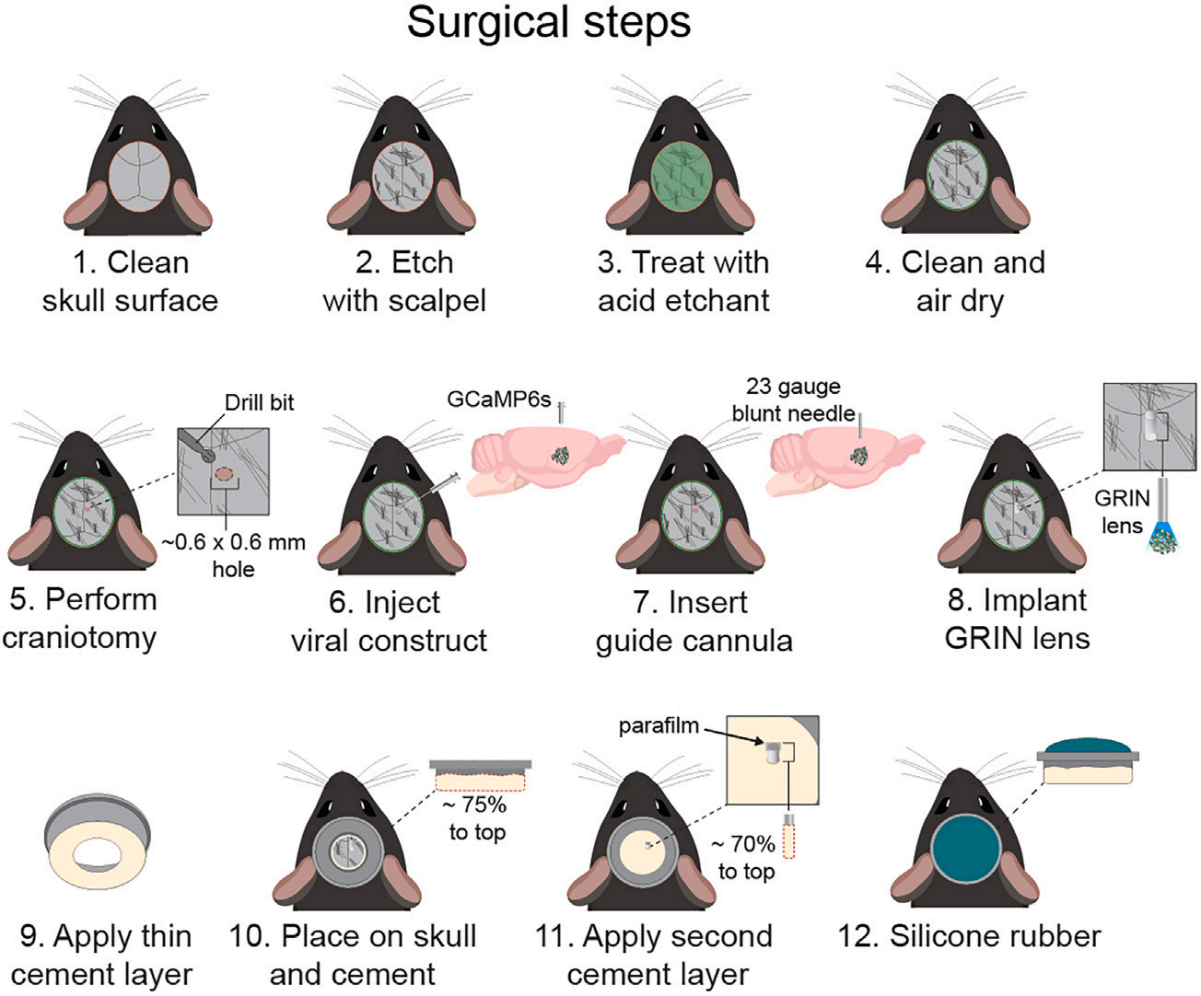
Steps for implantation of GRIN lens and surgical attachment of head-ring Diagrams that outline key surgical steps for GRIN lens implantation on attachment of head-ring.
  b. Gently score the skull with a scalpel to lightly roughen the surface (Figure 10: Step 2).
  c. Briefly apply one drop of Green Activator using a sponge or brush to the skull and leave on for 10 s.
    ***Note:*** The Green Activator in the Super-Bond C&B kit serves as an acid etchant composed of 10% citric acid and 3% ferric chloride. Brief application of this solution will microscopically roughen the skull to increase the strength of the head-ring to bond (Figure 10: Step 3).
  d. Rinse off etchant with sterile water.
  e. Thoroughly dry surface of skull (Figure 10: Step 4).
19. Define position of craniotomy.
  a. Level skull along the midline with bregma and lambda as reference points.
  b. Level the skull along the horizontal plane.

***Note:*** Once the position of the craniotomy is defined, we recommend calculating the dorsal ventral (DV) coordinates for the viral injection, GRIN lens, and guide cannula steps prior to advancing forward.

***Note:*** For viral injections we inject at two coordinates, the first is located 0.1 mm ventral of target site, and the second is located 0.1 mm dorsal of target site.

***Note:*** The GRIN lens is implanted 0.2 mm dorsal of target site, and the guide cannula is 80% of the GRIN lens DV.

16. Prepare the mouse for surgery.
  a. Weigh the mouse.
  b. Anesthetized with 3% isoflurane in induction chamber.
  c. Secure mouse in stereotaxic apparatus and adjust isoflurane to (1–1.5%).
  d. Insert rectal probe used to maintain body temperature of 37°C–38°C by using a feedback-controlled heating pad.
  e. Apply an extensive amount of sterile ophthalmic ointment to each eye.
    ***Note:*** Reapply ointment as often as needed and reduce light source to protect the eyes of the mouse.
  f. Inject sterile saline (0.9% NaCl solution) to prevent dehydration.
  g. Inject an immunosuppressant (Meloxicam, 5 mg/kg SQ)
17. Prepare surgical field.
  a. Apply hair-removal cream for ∼5 min.
    ***Note:*** Check instructions for application time, as it may differ across removal cream brands. In this protocol we use Nair hair-removal cream.
    ***Note:*** A second application of removal cream or another alternative hair removal approach (i.e. scissors or hair clippers) may be needed.
    **CRITICAL:** Keep hair-removal cream away from mouse eyes and whiskers.
  b. Remove the cream and scalp fur using cotton-tip swabs.
  c. Thoroughly disinfect scalp with several (3×) alternating washes of 10% povidone-iodine and 70% ethanol.
  d. Apply lidocaine and triple antibiotic ointments. Wait ∼5 min for topical agents to take effect.
  e. Make a vertical incision along the midline of the skull with a sharp and sanitized scalpel.
18. Prepare skull surface.
  a. Clean surface of skull with sterile saline and dry (Figure 10: Step 1).

**CRITICAL:** The working distance of GRIN lens differs across versions. It is important to adjust the DV coordinate appropriately based on the working distance associated with the GRIN lens model.

20. Create craniotomy (Figure 10: Step 5).
  a. Attach sterilized carbide burr drill bit to a micro drill secured to the stereotaxic frame.
    **CRITICAL:** Select a carbide drill bit that is slightly larger than the GRIN lens diameter (Figure 9B). It is important that the craniotomy is large enough to allow the GRIN lens to be lowered into position without getting scratched. But the craniotomy needs to be small enough to avoid the GRIN lens tilting during the cementing process.
  b. Navigate micro drill to location of craniotomy and carefully drill skull surface with smooth motions until bone is broken through.
    **CRITICAL:** Immediately stop drilling once bone is broken through to avoid damaging the brain or blood vessels.
21. Clean skull surface with sterile saline.
22. Inject viral construct containing GCaMP6s (Figure 10: Step 6).
  a. Secure a glass pipette filled with mineral oil to an injector that is fixed to one of the stereotaxic arms.
    ***Note:*** For preparation of glass pipettes for injection, we follow steps highlighted in these well written protocols.^11^^,^^12^
  b. Draw up ∼400 nL of virus with a micropipette and dispense on a clean piece of parafilm placed on one of the ear bars.
  c. Carefully navigate glass pipette to parafilm and draw up virus with injector without breaking the pipette.
  d. Zero coordinates of glass pipette at bregma.
  e. Locate pipette to coordinates of targeted brain regions.
  f. Lower glass pipette to first DV coordinate and wait 3 min.
  g. Inject 100 nL of virus at 20 nL/min rate (∼5 min) and wait an additional 5 min.
  h. Retract pipette slowly to second DV coordinate and wait 5 min.
  i. Perform a second injection of 100 nL at 20 nL/min rate. After virus injections, wait an additional 10 min before fully removing the glass pipette.
23. Lower guide Cannula (Figure 10: Step 7).
  a. Attach a sterile 23-gauge blunted needle to stereotaxic arm.
  b. Lower guide cannula to 80% of GRIN lens DV coordinate and immediately retract.

**CRITICAL:** This step is important as the blunted needle creates an initial path for the GRIN lens (Figure 9B). There will be blood since the needle will remove vascularized tissue. Due to this, it is necessary to clear the craniotomy of blood prior to lowering the GRIN lens. In addition, when lowering the GRIN lens, maintain hydration of the craniotomy with saline.

24. Lower Grin Lens (Figure 10: Step 8).
  a. Gently secure GRIN lens on stereotaxic arm using tweezers covered in soft material that will not scratch or damage the GRIN lens.
    ***Note:*** We found heat shrink to be an effective solution.
    ***Note:*** In addition, make sure the holder that will be used to lower the GRIN lens is also composed of material that will not scratch the GRIN lens.
  b. Slowly lower GRIN lens 2 mm dorsal to target site (i.e., −2.0 DV if target site is −4.0).
    ***Note:*** The following steps for lowering the GRIN lens occur in two phases. The first phase, the lens is slowly lowered in four intervals of 0.25 mm each. After each interval, a 2-min waiting period follows, making the total time for this phase approximately 10–12 min.
    ***Note:*** The second phase involves lowering the lens in ten intervals of 0.15 mm each. Before each 0.15 mm increment, the lens is first retracted by 0.05 mm. Like the first phase, each interval is followed by a 2-min waiting period, totaling about 25 min for this phase. Our protocol differs slightly from others by not aspiration brain tissue to create a column pocket for the GRIN lens. Instead, the 0.05 mm retraction before lowering aids in separating tissue, ensuring stable placement of the lens.
  c. Prepare Super-Bond cement kit as recommended by the company.
  d. Brush a thin layer of cement around the GRIN lens and wait 5–10 min to allow the cement to harden before removing the GRIN lens holder.
25. Attach head-ring to skull.
  a. Brush a thin layer of Super-Bond cement mixture onto the bottom of the etched head-ring.
  b. Carefully position the head-ring over the GRIN lens, ensuring that the y-plane of the lens aligns approximately at the center of the head-ring.
  c. Verify the alignment of the head-ring by visually inspecting its levelness.
    ***Note:*** Use the stereotaxic head bars as a reference to ensure proper alignment.
  d. Layer cement around the head-ring to further secure it in place (Figure 10: Step 9).
  e. Fill the middle of the head-ring with cement while also avoiding touching the top of the GRIN lens (Figure 10: Step 10).
  f. Wait for 10 min for the cement to fully dry.
    ***Note:*** An indicator to know when the cement is fully dried is that it will harden significantly.
  g. Place a small piece of parafilm on top of the GRIN lens to protect it from getting dirty (Figure 10: Step 11).
  h. Prepare silicone sealant as recommended by the company and layer on top of cement until the GRIN lens is fully covered by it (Figure 10: Step 12).
    ***Note:*** The sealant should solidify into rubber that can be easily removed between calcium imaging experiments without damaging the GRIN lens.
26. Provide postoperative care.
  a. Apply topical lidocaine and antibiotic ointments around the surgical site to mitigate pain and susceptibility to infection.
  b. Inject 0.1 mL of bupivacaine (0.5%, SQ) near the surgical site to reduce inflammation and pain.
  c. Gently remove the mouse from the stereotaxic apparatus and place it in a clean home cage located on top of a heating pad.
  d. Provide daily injections of meloxicam (5 mg/kg, SQ) for 2 days.
  e. Allow mouse to heal for 6 days post-surgery prior to habituation to head-fixation system.

***Note:*** It is possible to reuse head-rings after experiments without sacrificing mechanical stability in the head-fixation system. To remove the cement from the head-rings, they would need to be placed in acetone for several days or up to a week. The timeline is dependent on how easily the cement will detach from the head-ring to allow for proper cleaning and sanitation.

### Habituate to head-fixation system

**Timing: ∼6–8 days**

Pupillometry is a non-invasive measurement highly sensitive to changes in brain state and can be inadvertently influenced by stress and changes in environment.^13^^,^^14^^,^^15^ It is necessary to habituate mice to head-fixation to mitigate as many factors that could contribute to experimental bias, noise, or mouse-to-mouse variability during experiments.^16^ Here, we outline steps and a general timeline to properly habituate mice to the head-fixation system. We highly recommend recording the pupil and locomotion data during habituation as the number of days required for individual mice to achieve smooth mobility varies. In our experience, 6–8 days was a sufficient time frame for habituation. In addition, we recommend limiting habituation and experiments to 20 min per session.

***Note:*** Habituation and experiments should be conducted in a dark, quiet room. We place our wheel set-up in a soundproof box that encases the two-photon microscope. This box includes a red LED strip that illuminates the interior of the box for researchers to see when data is not being collected. Our microscope objective is configured on a motorized XYZ stage which fits above the locomotion wheel. The two-photon laser itself is positioned on a pneumatic optical table, outside of the sound-proof box. An optical path directs light into the microscope objective through a pinhole opening in the box.

27. Turn on all required devices.
28. Clean head-fixation system and box with disinfectant at the start of the session and each time mice are switched.
29. Fix mouse to the head-fixation system.
  a. Slide the head-ring attached to mouse skull into bottom headplate.
    ***Note:*** Ensure that the mouse head is straight and facing forward.
    **CRITICAL:** Prior to fixing the mouse to the system, ensure that a head-ring can seamlessly slide and be secured into the headplate (See troubleshooting: problem 4).
  b. Secure mouse into head-fixation system by placing top headplate over head-ring and securing with M3 screws.
    ***Note:*** The distance between the headplate and wheel for mice to walk comfortably depends on their size and age.^17^ In our experience, the distance for adult mice, independent of sex, to walk comfortably is approximately 3.175 cm, consistent with other protocol papers that utilize head-fixation systems.^17^^,^^18^
  c. Position LEDs ∼1.27 cm from mouse eye.
30. Open CaPuLeT GUI in MATLAB.
31. In the Setup tab, select the Serial port that is connected to Arduino.

***Note:*** The Status window will indicate if Arduino is connected, and the serial port is open.

32. Note any relevant information in the Experiment Info and Subject panels in the Setup tab.
33. Configure pupil camera in the Pupillometry tab (Figure 11).
  a. Under Device Connection panel, connect camera device.
    i. Select Refresh button to load cameras connected to the data acquisition computer.
    ii. Select FLIR camera and pixel format.
      ***Note:*** Once selected, the Status window will note whether the camera can be connected.
    iii. Connect pupil camera once the Status window indicates “Camera preview available”. Then turn on the camera preview.
  b. Set image brightness and LED intensities in Camera Settings panel.
    **CRITICAL:** For habituation experiments, the infrared LED allows visualization of the pupil in a low-light setting. During experimentation under the two-photon microscope, the infrared LED can be turned off since two-photon light illuminates the pupil of the mouse (See: Figure 13B).
    i. To constrict the pupil, adjust the Violet LED.
      **CRITICAL:** To capture pupil dynamics, it is necessary to constrict pupil to approximately less than or equal to 50% of its max pupil diameter.^15^
  c. (Optional) Select ROI in ROI Position panel to crop pupil image.
  d. Configure camera parameters for data collection.
    i. Select file format to save video file.
    ii. Select trigger to ensure video recording is aligned with other devices.
    iii. Select Configure Camera button to load all parameters for camera.

**Figure 11:**
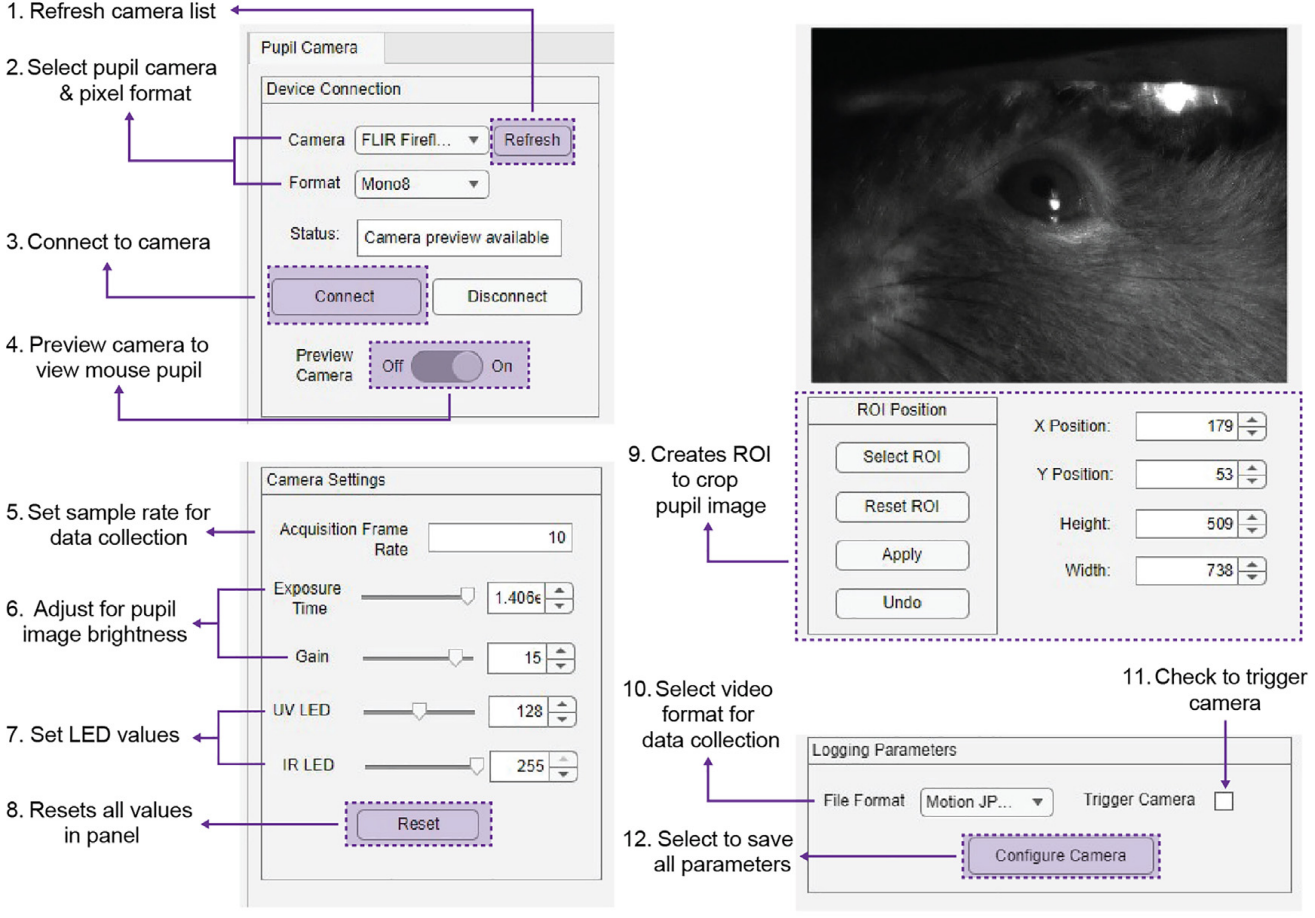
Parameters under the Pupillometry Tab in CaPuLeT GUI Layout of the panels that are in the CaPuLeT Pupillometry Tab to configure pupil camera for pupillometry experiments. Numbers indicate select configuration parameters.
34. Configure data collection of wheel data in “Locomotion-estimation” tab (Figure 12A).
  ***Note:*** To calculate velocity from the original output from the rotary encoder, we compute a ratio of cm per step using the circumference of the wheel and resolution of the rotary encoder (Figure 4C). The ratio is integrated in CaPuLeT to automatically calculate distance traveled that can be used to compute velocity.
  a. Set sample speed of wheel data in “Acquisition Speed” panel.
  b. Select file type to log wheel data in “Logging Parameters” panel.
  c. Select configure button to save all locomotion collection parameters.

**Figure 12:**
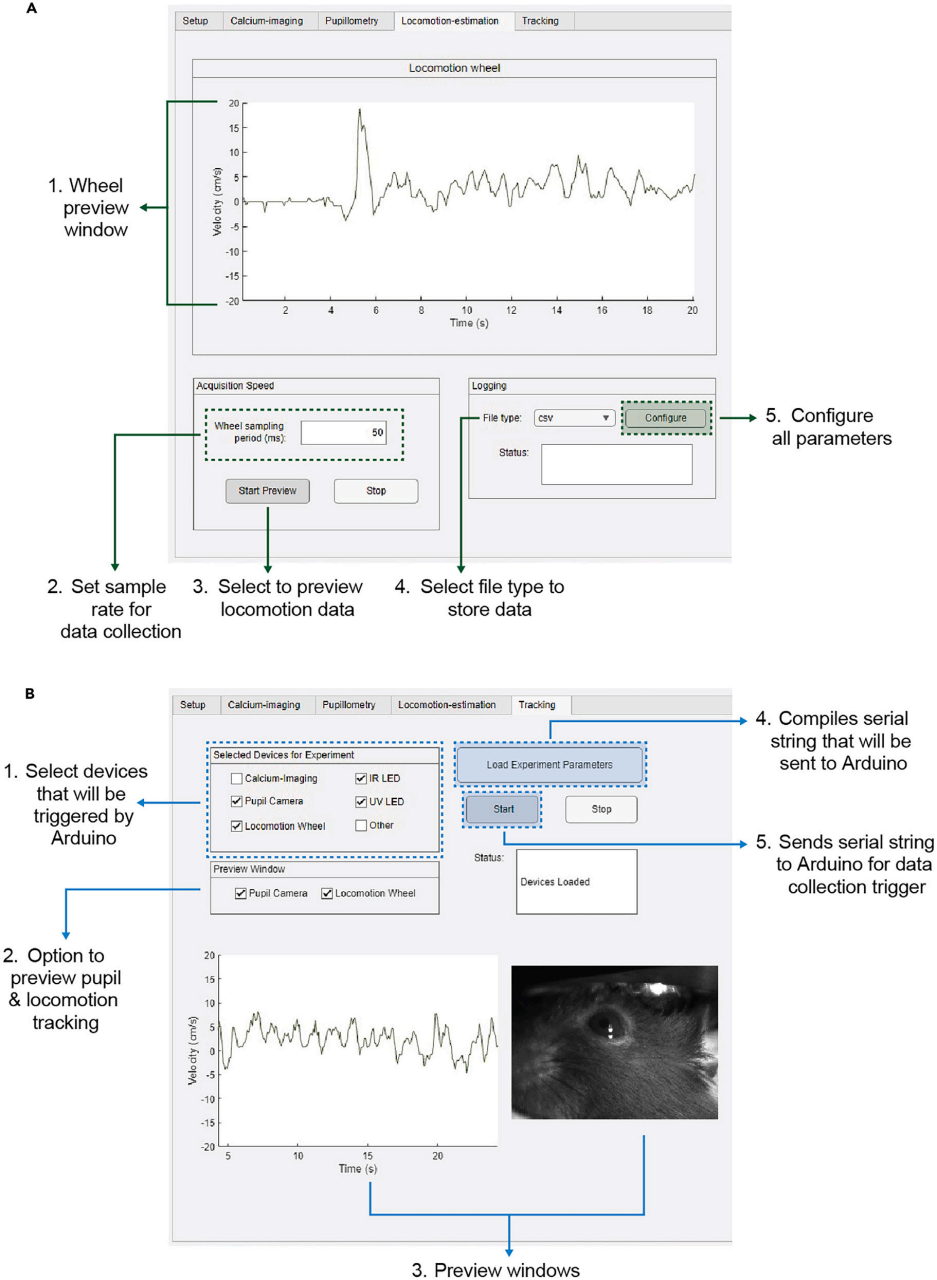
Locomotion-estimation and Tracking data parameters in CaPuLeT GUI (A) Example of CaPuLeT Locomotion-estimation Tab layout when an example mouse is running on the wheel. Numbers indicate preview of expected velocity output when an example mouse is moving on the wheel and specific parameters to configure sample rate and data logging. (B) Example of CaPuLeT Tracking Tab layout when habituating mice to wheel system. Numbers indicate general parameters to configure prior to data collection.
35. Initiate data collection in “Tracking” tab. (Figure 12B).
  a. Select devices for experiment in “Selected Devices for Experiment” panel.
    ***Note:*** For habituation experiments, the devices to select will be pupil camera, locomotion wheel, IR LED, and UV LED. If these devices were configured in the other tabs, these boxes would automatically be checked.
    ***Note:*** To preview the pupil camera and locomotion wheel during data collection, check these boxes. This will initially present empty windows that will show the wheel data and pupil video once the experiment begins.
  b. Select the Load Experiment Parameters button.
    ***Note:*** Load Experiment Parameters will generate the 9-digit serial string to be sent to Arduino to configure devices. Once that string is generated, the status window will state “Devices Loaded”.
  c. Select Start button to begin acquisition.
36. Leave mouse to habituate.

***Note:*** The timing for each habituation session depends on the length of the planned experiment. Our approach is to gradually increase the timing of each habituation session across several days. (i.e. Day 1 & 2: 5 min, Day 3 & 4: 10 min, Day 5 & 6: 15 min). The last two days of habituation should be for the duration of the planned experiment.

37. Assess locomotion at the beginning and then every other day of habituation to get accurate representation of proper locomotion across days.

### Extract pupil dynamics with DeepLabCut

**Timing: ∼3–5 days**

To extract pupil data from the pupillometry recordings, we follow steps highlighted in this well-documented protocol.^15^ In this protocol, Privitera et al.^15^ leverages DeepLabCut (DLC) to extract pupil dynamics from pupillometry video files. DLC is an open-source toolbox that utilizes deep learning algorithms trained to label relevant features from video files.^5^^,^^19^ Here, we broadly describe the main steps to extract pupil data using DLC.

38. Download and install relevant software to run DLC.
  a. Installation of DLC can be found here: https://github.com/DeepLabCut.
39. Train DLC network for pupil tracking.

**Figure 13:**
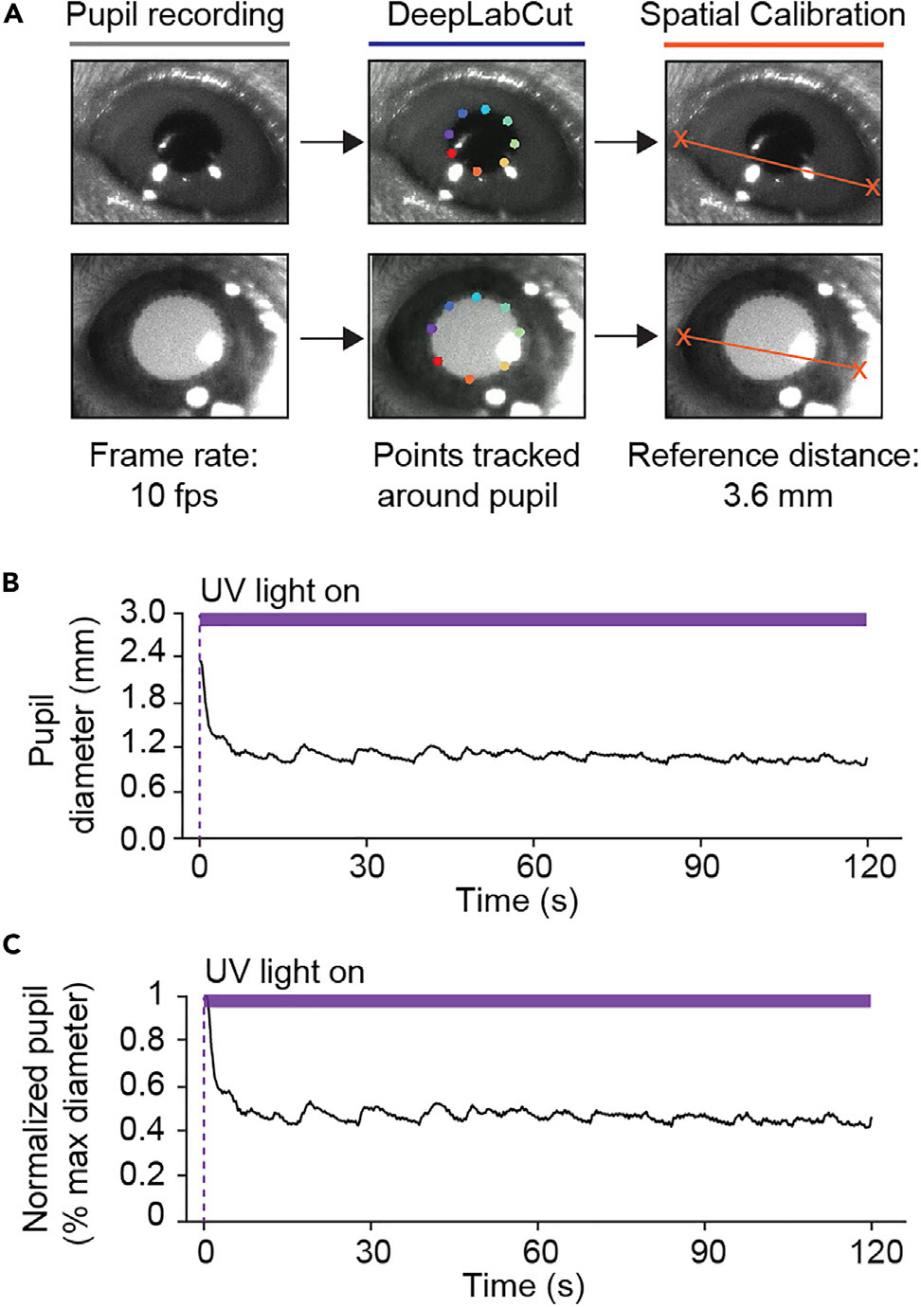
Extracted pupil measurements with DLC across habituation and calcium imaging experiments (A and B) Left: Example frames from pupil recording session during habituation (dark pupil in A) and two-photon calcium imaging experiments (white pupil in B); Middle: Results of DLC point tracking of pupil perimeter overlayed on original frame; Right: Location of spatial calibration points at the corners of the mouse eye and the reference distance between these points (3.6 mm). (C) Example trace of pupil diameter after conversion to metric representation from one mouse. (D) Example trace of pupil diameter normalized to max diameter.

***Note:*** For DLC to accurately estimate the pupil perimeter in the pupillometry recordings, a network needs to be trained on a diverse set of video frames with the pupil perimeter precisely annotated. We utilized the DeepLabCut GUI to extract frames from several different videos to create a diverse training dataset and we manually annotated the perimeter of the pupil with 8 different body part points (Figures 13A and 13B).

***Note:*** It is recommended to train a new network tailored for individual set-ups. The process to train a new network is highlighted in several videos listed on the DeepLabCut GitHub site as well as this thoroughly written protocol.^19^

40. Process video files with DLC.
  a. Open Anaconda and navigate to DLC environment.
    i. Select “Open with Python” option.
    ii. In the Python terminal, type the following command and enter:
      >import deeplabcut
    iii. To activate the GUI, type the following command and enter:
      >deeplabcut.launch_dlc()
  b. Once the GUI is launched, select “Load existing project”.
    i. Navigate to the file location that contains the trained network and import the configuration file “config.yaml”.
    ii. Navigate to the “Analyze videos” tab.
    iii. Specify video type as “.avi” and upload pupillometry videos.
      **CRITICAL:** DLC only accepts .avi or .mp4 video formats. Videos would need to be converted to those formats if the original video is outside those two types.
    iv. Indicate to have the data saved in the video file folder as ‘.csv’ files.
    v. Select “analyze videos” to process videos through DLC.
  c. Once all videos have been analyzed, navigate to the “Create Videos” tab to generate labeled videos.
    i. Select to plot body parts followed by “create labeled videos”.
      ***Note:*** Once the videos are created, the folder that contained the original pupillometry videos should be populated with ‘.csv’ files, one file per video, and mp4 videos containing points labeled around the perimeter of the pupil.
41. Identify spatial calibration points.

***Note:*** Spatial calibration points are needed to convert the data output from DLC to metric representation (Figures 13C and 13D). Here, we use the corner points of the mouse eye due to the distance between those points (∼3.6 mm in length) being a relatively consistent metric across mice (Figures 13A and 13B). It is possible to use other fixed objects of known distance that are in frame of the pupil recording as spatial calibration.^15^

### Acquisition of calcium imaging, pupillometry, and locomotion data

**Timing: ∼20 min/mouse**

Two-photon calcium imaging is a powerful approach to assess functionality of distinct neural ensembles across behavioral tasks during head-fixation.^7^^,^^10^ Here, we outline the additional steps needed to acquire data from all devices in the head-fixation system.

42. Turn on the two-photon laser 30 min prior to experimentation.
43. Open software that is used to control the two-photon microscope.

***Note:*** We use MATLAB based software known as ScanImage.

44. Place a shield on the objective of the two-photon microscope.

***Note:*** We place a shield on the objective to prevent the violet LED from reaching the photomultiplier tube (PMT) on the two-photon microscope. The shield is made using the same black adhesive contact paper placed on the locomotion wheel.

45. Secure mouse in head-fixation set-up as described in “habituate to head-fixation system” section.
46. Remove silicone sealant and clean GRIN lens.
47. Align focal plane for imaging of neurons.
  a. Place microscope above GRIN lens.
  b. Using software, turn on microscope using low power light and move microscope up and down until neurons are visible.

***Note:*** It is best to use low laser intensity (∼5–10% laser power) during alignment to prevent photobleaching.

**CRITICAL:** Laser safety eyewear that is protective against wavelengths in the IR domain (e.g., i.e.(700 nm - 1400 nm) with an appropriate Optical Density (OD) rating (OD 4+ or higher) must be worn to protect the experimenter’s eyes from the laser beam.

48. Increase laser intensity for experiment and configure other acquisition parameters.
49. Test trigger of laser from the CaPuLeT system under the “Calcium Imaging” tab.

***Note:*** The trigger delay time on the two-photon microscope software may need to be adjusted to account for the time the trigger signal from the Arduino will be received.

**Figure 14:**
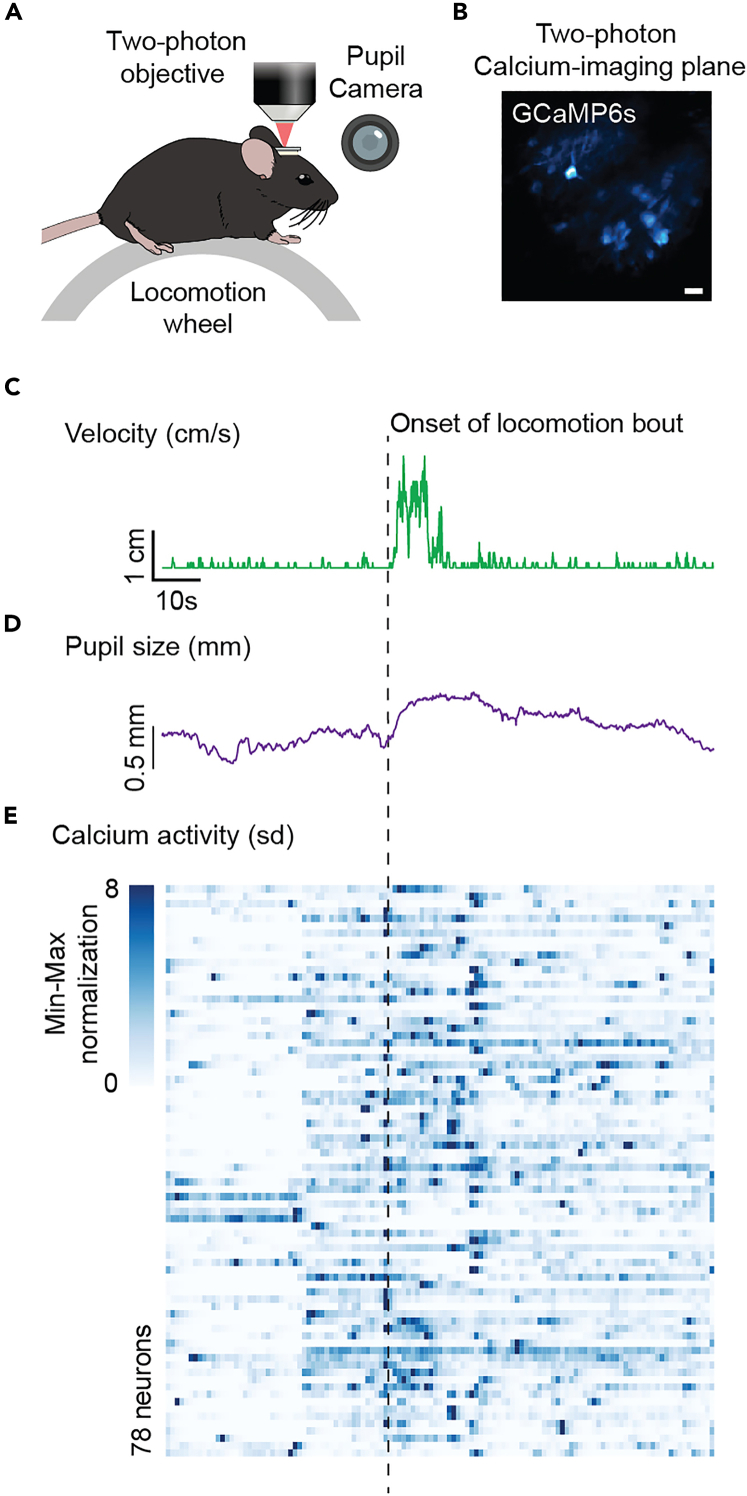
Synchronized data acquisition of locomotion, pupil, and calcium imaging dynamics (A) Schematic of experimental set-up. (B) Example image of neurons expressing calcium indicator GCaMP6s. Scale bar is 40 μm. (C and D) Sample traces of estimated locomotion activity and pupil diameter across a 3-min time interval. (E) Heat-map of neural activity across 78 neurons within a 3-min time interval.

50. Start data acquisition to record synchronized two-photon calcium imaging, pupillometry, and locomotion-estimated tracking (Figures 14A–14E).

## Expected outcomes

By utilizing the resources provided in GitHub and following the instructions outlined in this protocol, researchers will attain a head-fixation system equipped with a wheel apparatus, pupillometric tools, and device triggers for *in-vivo* calcium imaging devices. This integrated setup enables concurrent tracking of neural activity, pupil dynamics, and locomotion measurements in head-fixed mice. A custom Arduino shield printed circuit board maintains high temporal resolution across all electrical components, and the inclusion of a real time clock (RTC) module ensures synchronized data output during experiments. Furthermore, this system is engineered to seamlessly accommodate integration of other contemporary tools popularly used in neuroscience research, facilitating the monitoring or modulation of behavioral and neural outputs with minimal modifications required.

In addition, researchers will acquire the knowledge to perform the requisite steps for successful acquisition of calcium imaging, pupil, and locomotion data. To streamline the process of data acquisition, we designed a publicly available MATLAB graphical user interface: CaPuLeT. This GUI contains the necessary functions to trigger calcium-imaging software and to acquire pupillometry and locomotion data from an individual wheel set-up. For functionalities outside what is provided in the GUI, we hope that the current layout can serve as a base foundation for motivated researchers to easily tailor to their scientific research.

The recorded data from the head-fixation system includes video files for pupillometry and ‘.csv’ files for locomotion. The output file of videos processed with DLC is also ‘.csv’ files. These files contain columns of the x and y coordinates of all points labeled in pixels and the confidence score of that point being accurately detected. We utilize the spatial calibration points as a reference to convert the DLC data into absolute and normalized pupil measurements based on calculations outlined in this well-written protocol.^15^

Further, the recorded data from the wheel contains a count of pulse steps during mouse movement sampled at a desired frequency and as a function of time. To compute velocity from step count, the circumference of the wheel (∼67.83 cm) and the resolution of the incremental encoder (1024 pulse steps per revolution) is needed (Figure 4C). This information is used to calculate a ratio indicating distance moved (cm) per pulse step applied to extract velocity. CaPuLeT automatically calculates this conversion (Figure 12), and scripts would only need slight modification if a different wheel circumference or rotary encoder was used instead of the ones provided in this protocol. All scripts and README files for data acquisition and analysis are available on GitHub: https://github.com/UNC-optics/CaPuLeT. Further, any updates made to the CaPuLeT GUI will be available on GitHub.

## Quantification and statistical analysis

A custom R script is available on GitHub that contains several functions that when called, process pupil and locomotion data, and visualizes neural activity using a heat-map. For pupil analysis, the function “TransformDLC” takes in two parameters. The first is the x and y coordinates of the two spatial calibration points. The second is the ‘.csv’ file output from DLC that contains the x and y coordinates in pixels of all 8 points tracked. The distance between the spatial calibration points is calculated and then divided by the 3.6 mm reference distance to determine a ratio that will convert units in pixels to millimeters. The x and y coordinates of each point tracked in DLC are then multiplied by the conversion ratio and the distance between the points located across from one another is calculated. The average of these distances is used to represent pupil diameter as an absolute measurement (Figure 13C). In addition, this script incorporates thresholds to filter data based on the confidence score provided by DLC and minimal interpolation of x and y coordinates during periods of blinking.

Further, despite habituation, standardization of pupillometry systems and data collection practices, pupil size variability across mice is a well-reported challenge in neuroscience studies.^15^^,^^20^^,^^21^^,^^22^ Therefore, we also provide a function in our script to normalize pupil size based on the max pupil diameter across individual mice. Since the violet LED is triggered upon onset of data acquisition, we identify the max pupil diameter during the first couple of seconds of the experiment to transform our pupil diameter as a percentage (Figure 13D). We would like to also highlight that in addition to our code, there are other publicly available pipelines to seamlessly extract pupil data from DLC.^15^

To extract velocity from the data output in the head-fixation system, the function “TransformWheel” takes in the diameter of the wheel to perform simple computation to calculate circumference of the wheel and divide that value by the resolution of the encoder. We use this value to calculate distance moved and velocity from the data output. This function is integrated into CaPuLeT to perform automatic transformation of velocity and distance traveled (Figure 12). In addition, we also provide custom code that incorporates functions to quantify other locomotion metrics that can be computed. Calculations for these metrics are highlighted in this well-written protocol.^18^

For calcium imaging analysis, recordings were processed using the CaImAn pipeline.^23^ The extracted signals were adjusted (scaled) to account for variations in fluorescence intensities among cells by the standard deviation of a neuron’s fluorescence across a given trial. In R, the neural activity was binned in 1s intervals and displayed in the form of a heat-map that was not organized in a specific order (Figure 14E).

## Limitations

Although this head-fixation system was designed to integrate several systems relevant for neuroscience research and includes a user-friendly graphical user interface to simplify data collection, there are limitations that one must consider. First, this system restricts the maximum speed at which mice can run on the locomotion wheel, approximately 10 cm/s compared to the potential 40 cm/s speed observed in freely moving behavior. This speed impacts the interpretation of the type of locomotion behavior mice can engage in and limits certain locomotion studies.^24^ The design of the head-fixation bars only permits vertical adjustment of the mouse head without the capability for lateral movement, restricting the range of brain regions accessible for imaging. These brain regions include those that reside in the temporal lobe, such as auditory and visual cortices. The constraints on the mouse head, although useful for calcium imaging studies to reduce noise artifacts from motion, limit behavior assays that can be employed. These challenges may affect the breadth and applicability of certain findings, necessitating careful consideration in experimental design to mitigate these limitations.

Further, the Arduino shield was designed to allow researchers to perform modifications to the head-fixation system for other experiments that do not require two-photon microscopy. It is possible to upscale the number of head-fixation systems that Arduino can control and collect data from. However, the number of systems per Arduino is limited to 2. This is due to the number of interrupt pins available on the Arduino microcontroller, which are used to track locomotion. In addition, an Arduino can trigger multiple camera devices; however, the logging of video data can be computationally expensive. Prior to increasing the number of head-fixation systems, it is important to consider these limitations.

## Troubleshooting

### Problem 1

After connecting the pupil camera and additional camera to both the Arduino shield and the data acquisition computer, MATLAB is unable to detect either device (software setup, steps 2 and 7).

### Potential solution

The pupil camera (Teledyne FLIR) requires a USB3 driver available through the Spinnaker Software Development Kit (SDK). After downloading the SDK, the setup wizard will install the SpinView application on your device. SpinView will recognize the camera if it is connected to the data acquisition computer via a USB 3.1 type-A to micro-B cable, and automatically install the necessary driver. Drivers can be manually updated by selecting the “switch device driver” button after selecting a device. Additional third-party software is required to access the pupil camera through MATLAB’s image acquisition explorer. Installing both the IAT Support Package for GigE Vision Hardware and the IAT Support Package for GenICam Interface will allow MATLAB to access the pupil camera. The additional camera (The Imaging Source) requires a TIS UVC device driver which is available through the “Device Driver for USB Cameras” software. After downloading, your device will recognize the camera if it is connected via a USB 2.0 type-A to mini-B cable. Additional third-party software is required to access the additional camera through MATLAB’s image acquisition explorer. After downloading the “IC MATLAB Plugin for MATLAB”, a folder called “C:∖Program Files (x86)∖TIS IMAQ for MATLAB R2013b” will be installed on your device. Within this folder is a file called register_tisimaq.m, which is a MATLAB script that once run will register “TISImaq_R2013.dll” onto your device. This will allow MATLAB to access the additional camera.

### Problem 2

When the pupil camera is first initialized on SpinView, a default configuration file is saved that contains initial settings and configurations for the pupil camera. When the pupil camera is accessed outside of SpinView, the camera is configured based on this default file. Specific settings on SpinView can disable access for MATLAB to either detect or configure the pupil camera (software setup, steps 3 and 7).

### Potential solution

To access the pupil camera on SpinView, make sure to save these settings (an example of the SpinView settings is also available on GitHub).

Settings to save as device configuration on SpinView.

• Under Settings tab.
  ○ Enable Acquisition Frame Rate.
  ○ Disable Acquisition on boot. This will start acquisition of the camera when it is immediately connected to MATLAB, which will create issues with CaPuLeT.
• Under GPIO tab.
  ○ Trigger Selector should be “Frame Start”.
• Under features tab.
  ○ User Set Control should be set to “User Set 1”.

If other features not listed are unable to be accessed through MATLAB, it may be due to certain settings being selected. Look to Spinnaker SDK technical resources to resolve the issue. In addition, if the pupil camera is opened on SpinView, MATLAB will be unable to access the camera. If SpinView was opened when settings were changed at any point before experiment, take caution as these settings will automatically save and be configured in MATLAB. Additional troubleshooting tips will become available as we receive user feedback on GitHub.

### Problem 3

The incremental rotary encoder has a shaft diameter of 6 mm, while the linear motion shaft has a diameter of 5 mm. The shaft coupler supplied with the rotary encoder purchased from SparkFun is designed to accommodate two 6 mm diameter shafts. This mismatch in diameter can lead to a loose connection between the rotary encoder and the locomotion wheel, resulting in inaccurate locomotion measurements (step-by-step method details, step 1).

### Potential solution

The shaft coupler has four set screws which can be tightened to connect the rotary encoder shaft to the linear motion shaft. Given the millimeter difference in diameter between the linear motion shaft and the shafter coupler, one to two layers of electrical tape wrapped around the end of the linear motion shaft will provide a tighter fit. The set screws of the shaft coupler will stabilize after the electrical tape is added.

### Problem 4

The files that contain the dimensions used to machine the headplates for fixation are precisely designed to ensure a seamless fit for the head-ring. When ordering parts from a CNC machining service, it is important to verify the lowest tolerance accepted by the company. The tolerance is the specified amount of variation in the dimensions of a part that the company considers acceptable when machining parts to specifications. Tolerance ranges can vary substantially across different companies, and several companies offer different tolerance levels. The lowest tolerance accepted from the manufacturer we used was ± 0.0127 cm. Even at this level of manufacturing precision, we noticed some instances where the bottom headplate was slightly too tight for the head-ring, and we experienced resistance when sliding the head-ring in place (step-by-step method details, step 29).

### Potential solution

We advise not adjusting the dimensions of the bottom headplate or head-ring listed in the CAD files to accommodate the issue above. This will likely shift how secure the mouse will be in the head-fixation system and potentially lead to undesired head movement during experiments. Rather, we suggest manually sanding the bottom headplate while routinely assessing the head-ring fit. Another option is to manufacture a few head-rings with the same metal used to machine the headplate (Stainless steel 304) to stretch the bottom headplate. We successfully used this strategy to increase the dimensions of the headplate until the head-ring can easily fit. It is important that the head-rings used for surgery are not used for this process. The metal machined for those specific head-rings is weaker than the headplate metal and will bend, impacting the durability across time.

## Resource availability

### Lead contact

Further information and requests for resources should be directed to and will be fulfilled by the lead contact and corresponding author, Jose Rodriguez-Romaguera (jose_rodriguezromaguera@med.unc.edu).

### Technical contact

Technical questions on executing this protocol should be directed to and will be fulfilled by the technical contact and corresponding author, Nicolas C. Pégard (pegard@unc.edu).

### Materials availability

The CAD files for the custom locomotion wheel, head-fixation system, head-rings, rotary encoder adaptor, and the fabrication files for the Arduino shield printed circuit board are available on GitHub: https://github.com/UNC-optics/CaPuLeT.

### Data and code availability

The MATLAB, Arduino, and R scripts generated during this study are available on GitHub: https://github.com/UNC-optics/CaPuLeT. Example pupillometric and locomotion data is available upon request to the technical contact and corresponding author, Nicolas C. Pégard (pegard@unc.edu).

## Acknowledgments

The authors would like to thank Randall L. Ung and Jose L. Rios for technical assistance with the head-fixation system, Dr. Andrea Giovannucci’s Lab for providing files for initial iterations of the 3D printed resin ramp, and Ayden L. Ring for helpful comments on the manuscript. This work was supported by grants from the 10.13039/100000001National Science Foundation (DGE-1650116, M.M.O.-J.), the 10.13039/100000011Howard Hughes Medical Institute Gilliam Award (GT15752, M.M.O.-J. and J.R.-R.), the Initiative for Maximizing Student Development (R25GM055336, J.T.-V.), the Royster Fellowship (V.R.C.), the 10.13039/100000861Burroughs Wellcome Fund (N.C.P.), the 10.13039/100000879Alfred P. Sloan Foundation (N.C.P.), a Beckman Young Investigator Award (N.C.P.), the NC TraCS Institute (J.R.-R. and N.C.P.), the 10.13039/100018784Foundation of Hope (J.R.-R.), the 10.13039/100000874Brain and Behavior Research Foundation (J.R.-R.), the 10.13039/100001201Kavli Foundation (J.R.-R.), the 10.13039/100001391Whitehall Foundation (J.R.-R.), and the 10.13039/100000025National Institute of Mental Health (R01MH132073, J.R.-R.).

## Author contributions

Conceptualization, M.M.O.-J., J.T.-V., N.C.P., and J.R.-R.; iteration and finalization of head-fixation system, M.M.O.-J., J.T.-V., R.A.A., and N.W.M.; code generation, M.M.O.-J., V.R.C., and N.C.P.; MATLAB GUI, M.M.O.-J. and N.C.P.; data acquisition used in figures, M.M.O.-J. and V.R.C.; resources, J.T.-V. and E.M.M.; GitHub organization, J.T.-V. and S.M.H.; writing – original draft, M.M.O.-J., J.T.-V., and S.M.H.; writing – review and editing, M.M.O.-J., J.T.-V., N.C.P., and J.R.-R.; figure preparation, M.M.O.-J., J.T.-V., and S.M.H.; supervision, N.C.P. and J.R.-R.

## Declaration of interests

N.C.P. and J.R.-R. are co-founders of a company (Carolina Instruments, LLC) with a potential commercial interest in the technology presented in this manuscript.

## Declaration of generative AI and AI-assisted technologies in the writing process

During the preparation of this work, the authors used Microsoft Copilot to generate an image depicting a mouse on a wheel overlooking another mouse for the CaPuLeT MATLAB graphical user interface. After using Microsoft Copilot, the authors reviewed and edited the content as needed and take full responsibility for the content of the publication.
